# Hox gene–specific cellular targeting using split intein Trojan exons

**DOI:** 10.1073/pnas.2317083121

**Published:** 2024-04-11

**Authors:** Fengqiu Diao, Deeptha Vasudevan, Ellie S. Heckscher, Benjamin H. White

**Affiliations:** ^a^Laboratory of Molecular Biology, Section on Neural Function, National Institute of Mental Health, NIH, Bethesda, MD 20892; ^b^Department of Molecular Genetics and Cell Biology, University of Chicago, Chicago, IL 60637

**Keywords:** *Drosophila*, genetic access, neural circuit-mapping, development, cell type

## Abstract

The Trojan exon method, which makes use of intronically inserted T2A-Gal4 cassettes, has been widely used in *Drosophila* to create thousands of gene-specific Gal4 driver lines. These dual-purpose lines provide genetic access to specific cell types based on their expression of a native gene while simultaneously mutating one allele of the gene to enable loss-of-function analysis in homozygous animals. While this dual use is often an advantage, the truncation mutations produced by Trojan exons are sometimes deleterious in heterozygotes, perhaps by creating translation products with dominant negative effects. Such mutagenic effects can cause developmental lethality as has been observed with genes encoding essential transcription factors. Given the importance of transcription factors in specifying cell type, alternative techniques for generating specific Gal4 lines that target them are required. Here, we introduce a modified Trojan exon method that retains the targeting fidelity and plug-and-play modularity of the original method but mitigates its mutagenic effects by exploiting the self-splicing capabilities of split inteins. “Split Intein Trojan exons” (siTrojans) ensure that the two truncation products generated from the interrupted allele of the native gene are trans-spliced to create a full-length native protein. We demonstrate the efficacy of siTrojans by generating a comprehensive toolkit of Gal4 and Split Gal4 lines for the segmentally expressed Hox transcription factors and illustrate their use in neural circuit mapping by targeting neurons according to their position along the anterior–posterior axis. Both the method and the Hox gene–specific toolkit introduced here should be broadly useful.

## Significance

The brain has hundreds of cell types, many of unknown function. Determining the functions of all brain cell types requires methods for selectively monitoring and perturbing their activity. This is best accomplished by genetic methods, but these methods frequently disrupt the function of the native genes whose regulatory information is co-opted to achieve cell type–specific targeting. Here, we present a method for targeting specific cell types that limits disruption of the genes used to gain genetic access to them. We demonstrate the method’s utility by using it to identify and manipulate neurons that express different Hox transcription factor genes and are therefore located in anatomically distinct regions along the anterior–posterior axis of the fly nervous system.

**Fig. 1.**
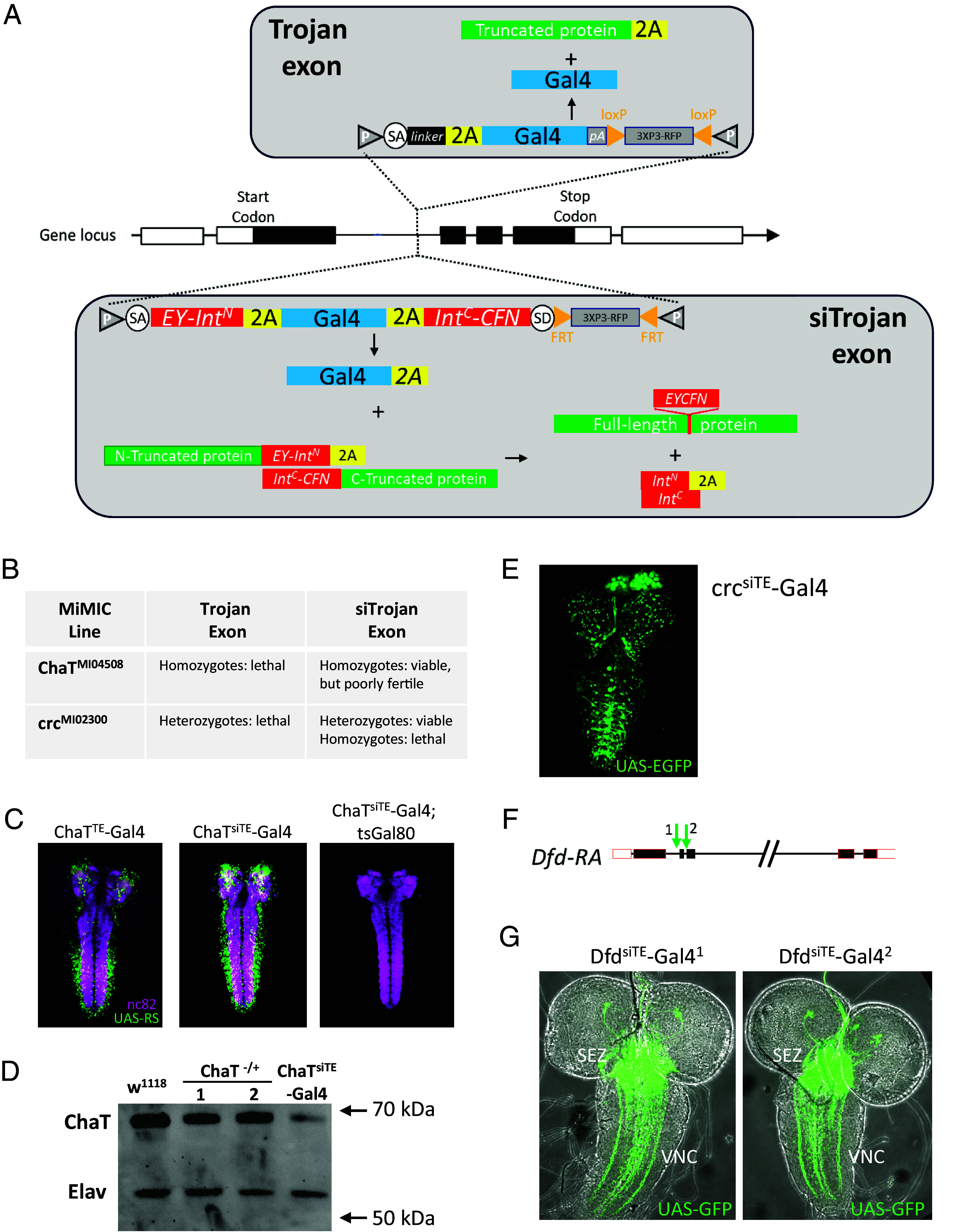
The architecture and application of split intein Trojan exons. (*A*) Schematic illustrating the original Trojan exon strategy (*Top*) and the new split intein Trojan exon strategy (*Bottom*). In both cases, the synthetic exon is inserted into an intron separating two coding exons of a native gene. The original Trojan exon produces the Gal4 protein and a truncation product of the native protein. The siTrojan exon technique produces three translation products: the Gal4 molecule fused at its C terminus to a T2A peptide and two truncated fragments of the native protein. The N-terminal fragment is fused at its C terminus to the Cfa^N^ split intein moiety (EY-Int^N^) and a T2A peptide; the C-terminal fragment is fused at its N terminus to the Cfa^C^ split intein moiety (Int^C^-CFN). These two truncated products of the native protein are ligated by split intein-mediated trans-splicing. (*B*) Driver lines made by inserting siTrojan-Gal4 constructs into the *ChaT* and *crc* genes by ΦC31-mediated MiMIC cassette exchange have reduced lethality compared to classical Trojan-Gal4 lines made via insertions into the same MiMIC sites. (*C*) Larval CNS expression of ChaT-Gal4 lines made as in (*B*) using classical Trojan exon technology (*Left*, ChaT^TE^-Gal4) and siTrojan exon technology (*Middle*, ChaT^siTE^-Gal4). The ChaT^siTE^-Gal4 line remains sensitive to tsGal80 inhibition at the restrictive temperature of 31 °C (*Right*) despite the T2A tail on Gal4. Green, UAS-RS (RedStinger) expression; magenta, nc82 neuropil staining. (*D*) Western blot showing full-length ChaT protein in CNS of flies homozygous for the ChaT^siTE^-Gal4 insertion. 2X CNS equivalents were loaded in all lanes for flies of either the control (w^1118^) or experimental (ChaT^siTE^-Gal4/ChaT^siTE^-Gal4) genotypes and immunostained for ChaT protein. For comparison, results are shown for hemizygous flies with exonic insertions into the same ChaT intron (1: MiMIC line, ChaT^MI04508^/TM3, Ser; 2: the original Trojan exon line, ChaT^TE^-Gal4/TM6B). MW markers are as indicated and immunostaining of the panneuronal protein Elav is included as a loading control. (*E*) L3 larval CNS expression of the crc^siTE^-Gal4 line created by conversion of MiMIC line MI02300 using a siTrojan-Gal4 construct. (*F*) Intronic sites of CRISPR/Cas9-mediated insertion (green arrows) of Trojan-Gal4 and siTrojan-Gal4 constructs into the *Deformed* gene. Insertions of the original Trojan exon produced a viable line only at site 1, siTrojan exon insertions at both sites 1 and 2 produced viable lines with robust Gal4 expression in the expected patterns of the *Dfd* gene: (*G*) Larval CNS expression of Dfd^siTE^-Gal4 lines produced by siTrojan exon insertion as in (*F*). Strong labeling is seen in cell bodies in the SEZ and their axons projecting to the ventral nerve cord (VNC).

Synthetic exons have been adapted for performing a broad range of genetic and cellular manipulations since their introduction as part of the versatile MiMIC toolkit for disrupting gene function in *Drosophila* (1–3). One important application of this technology has been to gain genetic access to targeted cells by inserting synthetic exons encoding T2A-Gal4 cassettes into the introns of cell type-specific genes (1, 4–6) (Fig. 1 *A*, *Top*). Because Gal4 can be used to express a wide range of transgenes, from gene-specific RNAis to optogenetic actuators of cellular excitability, this method offers considerable control over cellular function. Synthetic exons encoding T2A-Gal4 (or other transcriptional activators) have thus been called Trojan exons by analogy to the Trojan horse.

Trojan exons, like the swappable integration cassettes of MiMIC constructs, are fully modular, with T2A-Gal4 easily exchanged for other transcriptional activators such as LexA or Split Gal4 hemidrivers by ΦC31 cassette exchange. Insertion of Trojan exons into the introns of targeted genes is also readily accomplished with CRISPR/Cas9 using constructs such as TGEM (Trojan Gal4 Expression Module) (4) or CRIMIC [CRISPR-mediated integration cassette (1)]. While the modularity and ease of targeting of Trojan exons has facilitated their use, efforts to create Trojan exon lines specific for certain genes, most notably essential transcription factors, have been compromised by developmental lethality (4). For at least some of these transcription factors, Trojan exon insertion likely generates a truncated protein product with dominant-negative function. For example, the developmentally important transcription factor Abrupt heterodimerizes with other transcription factors, such as Taiman, via a BTB domain (7), and this domain, but not the Abrupt DNA-binding domain, was included in the predicted truncation product of the Trojan exon insertion into the *abrupt* gene. To mitigate such potential mutagenic effects of Trojan exons, while retaining the simplicity and modularity of the original method, we created a modified version in which the truncation products self-repair using the trans-splicing capabilities of split inteins (Fig. 1 *A*, *Bottom*).

Split inteins come in N- and C-terminal halves (i.e., Int^N^ and Int^C^), each of which can be incorporated into a separate protein (8). Each half is functionally inert, but when expressed together, the two halves will associate and covalently ligate the two proteins to which they are fused, excising themselves from the final product. Numerous examples of both natural and synthetic split inteins have been characterized (9, 10). They vary widely in their efficacies and splicing rates, which typically depend strongly on the identity of amino acids flanking the splice sites (11). Among the fastest naturally occurring split inteins is “Npu” of the cyanobacterium *Nostoc punctiforme*, which is responsible for generating the α-subunit of DNA Polymerase III (DnaE). A variant, derived from the consensus sequence of Npu and 72 homologous DnaE inteins and displaying high stability and fast splicing kinetics in a variety of protein contexts, was introduced by Stevens et al. (12). Here, we incorporate this optimized split intein, called Cfa for “Consensus fast,” into a Trojan exon to catalyze the splicing of the two native protein fragments generated by interruption of the native gene.

We demonstrate the efficacy of this split intein Trojan exon (siTrojan) technology by applying it to the genes encoding the developmentally essential class of Hox transcription factors. Hox genes were initially discovered in the fruit fly, where they are expressed in a segmental fashion along the anterior–posterior (A-P) axis of the body and nervous system (13, 14). They have subsequently been found to play critical roles in A-P patterning in all bilaterian animals and have generated considerable interest recently because of their proposed roles in patterning motor circuits underlying behavior in both flies and mice (15–18).

The importance of understanding how Hox genes specify diversity of both cellular identity and circuit function across the A-P axis of the CNS highlights the need for methods for gaining genetic access to the cells that express them. In *Drosophila*, driver lines with varying specificity for each of the eight Hox genes have been produced over the last two decades using a variety of methods (*SI Appendix*, Table S1). Notably, however, no Hox gene–specific Gal4 driver lines have been reported that use Trojan exon technology. However, alternative approaches to achieving gene-specific expression have been used to produce drivers for four Hox genes [*proboscipedia* (*pb*), *Deformed* (*Dfd*), *Sex combs reduced* (*Scr*) and *Antennapedia* (*Antp*)] (19, 20). Hox gene–based hemidrivers for intersectional targeting using the Split Gal4 method (21) have also been made for three Hox genes: *Dfd*, *Ultrabithorax* (*Ubx*), and *Abdominal-B* (*Abd-B*) (20, 22).

Here, we systematically create a comprehensive set of siTrojan Gal4 drivers and Split Gal4 hemidrivers for the eight *Drosophila* Hox genes. We show that these lines broadly recapitulate known patterns of Hox gene expression, and we illustrate their utility in the neural study of behavior by targeting subsets of motor neurons according to their position along the A-P axis and by parsing neurons within a given Hox gene domain according to their neurotransmitter usage. Beyond significantly expanding the toolkit of Hox-specific reagents in the fly, the siTrojan exon method introduced here represents an important addition to the available techniques for making gene-specific drivers. The method should be applicable in other species and generally useful for gaining genetic access to cell types defined by their expression of specific genes or gene combinations (23, 24). Gaining such access is an increasingly important challenge as transcriptomic studies continue to generate cell types that demand anatomical and functional characterization (25).

## Results

### The Cfa Split Intein Reconstitutes the Function of Split Proteins in *Drosophila* Cells.

To capitalize on the gene-specific transgene expression achievable by the Trojan exon method while mitigating that method’s mutagenicity, we introduced two principal changes. First, we replaced the polyadenylation signal following the Gal4 sequence of the original Trojan exon construct with the sequence encoding a second T2A peptide (Fig. 1*A*, compare bottom vs. top). This change ensures not only the independent production of Gal4 but also the translation of the C-terminal fragment of the native gene located downstream of the Trojan exon. Since this C-terminal fragment is translated separately from the N-terminal fragment, however, a means of joining the N- and C-terminal fragments to form the intact, full-length protein is required. To permit this, we introduced a second change, placing split intein moieties on either side of the 2A-flanked Gal4 gene.

We incorporated the N- and C-terminal halves (Cfa^N^ and Cfa^C^) of the optimized Cfa split intein developed by Stevens et al. (12) into the design of our modified Trojan exon so that they would catalyze splicing of the two native protein fragments generated by introduction of the exon (Fig. 1 *A*, *Bottom*). To ensure optimal performance in diverse protein contexts we also elected to retain in our design amino acid residues flanking the native splice site of Npu, the naturally occurring split intein from which Cfa was derived (26). These residues are EY for Cfa^N^ and CFN for Cfa^C^ (*SI Appendix*, Fig. S1*A*). Depending on translation reading frame, this necessarily results in a six- or seven-amino-acid insertion into the protein encoded by the gene into which the Trojan exon is inserted (*SI Appendix*, Fig. S1*B*).

To test the ability of the Cfa split intein to catalyze trans-splicing in *Drosophila* cells, we created two constructs, which together encode a split LexA transcription factor (*SI Appendix*, Fig. S2*A*). The first construct encodes the LexA DNA-binding domain fused to EY-Cfa^N^, and the second encodes Cfa^C^-CFN fused to a p65AD transactivation domain. *Drosophila* S2 cells transfected with either construct alone failed to drive expression of a LexAop-6XmCherry reporter, but strong reporter expression was observed when cells were transfected with both constructs (*SI Appendix*, Fig. S2 *B* and *C*).

### Trojan Exons Incorporating the Cfa Split Intein Limit the Mutagenic Effects of Protein Truncation.

To directly test the efficacy of the split intein strategy in vivo, we used ΦC31-mediated cassette exchange to insert a siTrojan-Gal4 construct into an intronic MiMIC site (MI04508) in the *choline acetyl transferase* (*ChaT*) gene. We previously showed that insertion of a traditional Trojan-Gal4 construct into this site results in a ChaT-specific Gal4 driver that is homozygous lethal for the inserted construct due to the mutagenic effects of truncating the *ChaT* gene (Fig. 1*B*) (4). In contrast, the siTrojan-Gal4 insertion into the same site produced animals that were homozygous viable but retained the same robust gene-specific Gal4 expression of the traditional Trojan ChaT^TE^-Gal4 driver (Fig. 1*C*). Western blot analysis demonstrated that homozygotes generated full-length ChaT protein, albeit at reduced levels compared with controls (Fig. 1*D*). Homozygotes were also observed to be poorly fertile, suggesting incomplete restoration of gene function. Overall, however, these results establish the in vivo efficacy of the Cfa split intein and demonstrate the reduced mutagenicity of siTrojan constructs compared with the original Trojan exons.

One difference between the Gal4 molecules translated from the two types of Trojan exons is that the siTrojan Gal4 bears a T2A peptide at its C terminus. The Gal4 inhibitor, Gal80, binds to the C terminus of Gal4 and is often used to modulate Gal4 activity in vivo (27). We used the temperature-sensitive Gal80 allele, tsGal80 (28), to determine whether the ChaT^siTE^-Gal4 molecule retains its sensitivity to Gal80 and found that Gal80 inhibition remained robust (Fig. 1 *C*, *Right*).

In a second test of efficacy, we inserted a siTrojan-Gal4 construct into an intronic MiMIC site in the gene encoding the *Drosophila* ATF4 homolog, *cryptocephal* (*crc*). We previously reported that a traditional Trojan Gal4 construct was unable to generate viable transformants when targeted to this site (4). This is consistent with the dominant lethality associated with a *crc* deletion mutant [R6; (29)] that is predicted to yield the same truncation product as the Trojan exon insertion. The insertion of the siTrojan-Gal4 construct, however, yielded healthy and viable transformants (Fig. 1*B*) and robust Gal4 expression (Fig. 1*E*). Again, however, the siTrojan-Gal4 insertion was homozygous lethal indicating some persistent impairment of function.

As a final test of efficacy, we compared the effects of inserting siTrojan-Gal4 and traditional Trojan-Gal4 exons (i.e., “TGEM” constructs) into the same two intronic sites in the *Deformed* (*Dfd*) gene (Fig. 1*F*). Dfd is a Hox transcription factor, which in the central nervous system has expression restricted to neurons of the gnathal ganglia within the subesophageal zone (SEZ) (19, 20, 30). While only one of the two TGEM constructs produced viable transformants, both siTrojan-Gal4 constructs did so. Both expressed strongly in the SEZ of larval nervous systems (Fig. 1*F*), and morphometric analysis of gnathal ganglia volume revealed no overt changes as a result of siTrojan-Gal4 expression (*SI Appendix*, Fig. S3 *B* and *C*). The TGEM-generated line also expressed in the SEZ, but unexpectedly exhibited ectopic expression in some VNC neurons (*SI Appendix*, Fig. S3*A*).

We conclude that siTrojan exons represent an effective method for generating gene-specific Gal4 lines in *Drosophila*. Our results demonstrate that the mutagenic effects of siTrojans are much less deleterious than those occasioned by the original Trojan exon technology. In addition, the T2A peptide attached to the C terminus of Gal4 does not impair Gal4 activity or its sensitivity to Gal80.

### Generation of Gene-Specific Driver Lines for All *Drosophila* Hox Transcription Factors.

*Dfd* is one of eight *Drosophila* Hox genes, the expression of which span distinct regions of the A-P axis. The mechanisms that regulate the regional specificity of Hox gene expression are complex, as are the interactions between Hox proteins and their diverse transcriptional partners (31, 32). They therefore represent a challenging set of genes to make drivers for. As a test of the siTrojan technology, we therefore sought to produce a complete set of siTrojan Hox Gal4 lines that could be used to generate further Hox-specific lines expressing other effectors.

**Fig. 2.**
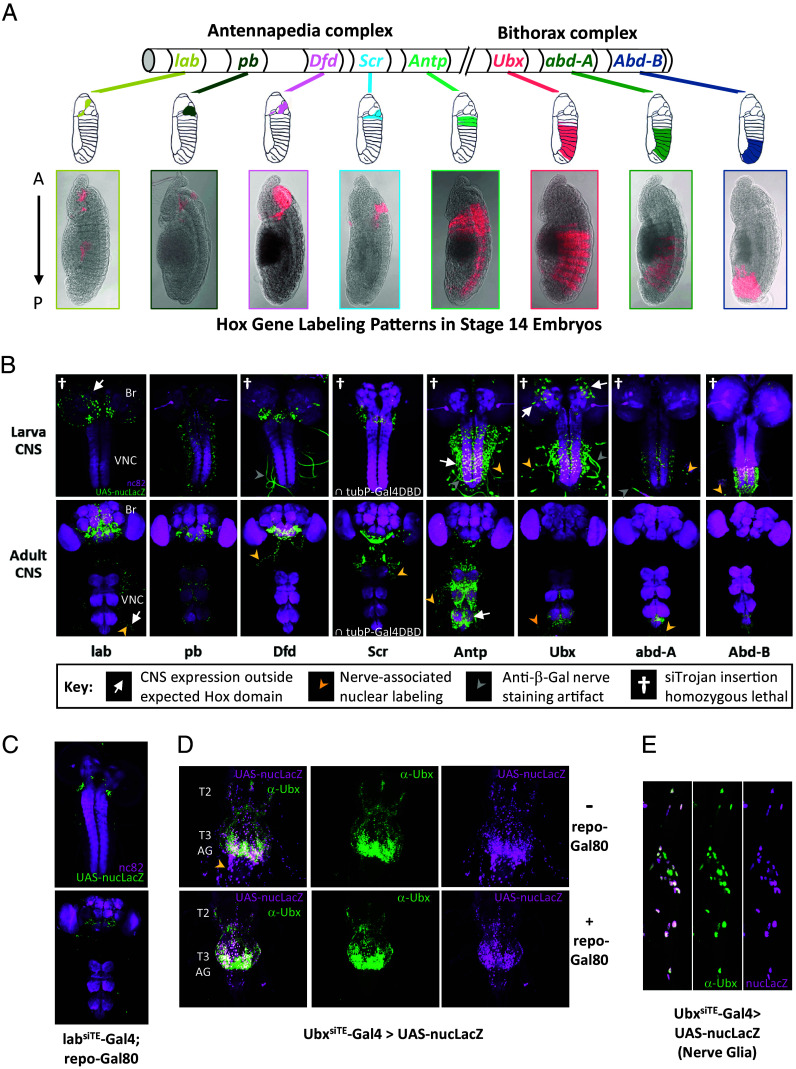
Embryonic and nervous system expression of Hox Gene–specific siTrojan Gal4 drivers. (*A*) Expression of the Hox^siTE^-Gal4 drivers (p65AD∩tubP-Gal4DBD for *Scr*) varies across the A-P axis in stage 14 embryos (*Bottom* images). The patterns are consistent with those observed in previous studies (schematics from Hughes and Kaufman (13) used with permission; License 5645480954289) and change progressively with Hox gene position along the 3R chromosome. The two complexes of Hox genes on 3R are indicated. Red, UAS-6XmCherry. “Saturation” and image “Exposure” in the red channel were adjusted in Photoshop (to settings of 25 and 2.0, respectively) to enhance visualization. (*B*) CNS expression of the Hox-specific drivers in third instar larvae (*Top*) and adults (*Bottom*). Br, brain; VNC, ventral nerve cord. Here and subsequent panels: green, UAS-nucLacZ visualized with anti-beta-galactosidase antibody; magenta, nc82 neuropil labeling. Arrows and arrowheads, unexpected labeling; crosses, patterns of drivers that are homozygous lethal, as indicated in key. (*C*) CNS expression of the lab^siTE^-Gal4 driver in third instar larvae (*Top*) and adults (*Bottom*) is limited to the tritocerebral region of the brain when repo-Gal80 is used to block Gal4 activity in glia. (*D*) Cells in the abdominal nerves of the VNC (yellow arrowhead) are labeled by the Ubx^siTE^-Gal4 driver in the absence (*Top*), but not in the presence (*Bottom*) of repo-Gal80, indicating that they are glia. Thoracic neuromeres T2-T3 and abdominal ganglia (AG) are indicated. (*E*) Glia cells of the abdominal nerves labeled by Ubx^siTE^-Gal4 are immunopositive for anti-Ubx antibodies, indicating that the driver expression in these cells is not ectopic.

To complement the Dfd^siTE^-Gal4 lines already made, we targeted siTrojan-Gal4 insertions to intronic sites in the remaining seven Hox genes (*SI Appendix*, Fig. S4) and obtained viable transformants for *labial* (*lab*), *proboscipedia* (*pb*), *Antennapedia* (*Antp*), *Ultrabithorax* (*Ubx*), *abdominal-A* (*abd-A*), and *Abdominal-B* (*Abd-B*). Only for the *Sex combs reduced* (*Scr*) gene were we unable to successfully obtain viable transformants with siTrojan-Gal4 constructs. Because *Scr* has a single coding intron, and all siTrojan-Gal4 insertions into this intron will produce the same translation products, we did not test additional PAM sites. However, we were able to obtain viable transformants for this gene using an alternative siTrojan exon encoding the Split Gal4 transcriptional component, zip^+^-p65AD— the transcription activation domain of the p65 transcription factor. Combining Scr^siTE^-p65AD with a complementary and ubiquitously expressed tubP-Gal4DBD Split Gal4 component allows selective reconstitution of Gal4 activity in the *Scr* expression pattern, which can be compared with the Gal4 activity of the other Hox gene–specific lines (Fig. 2 *A* and *B*).

An iconic feature of *Drosophila* Hox genes is that they are expressed along the A-P axis of the body and brain in the same order that they are aligned along the right arm of the third chromosome (14). At stage 14 of embryonic development, expression of the eight Hox gene–specific siTrojan drivers broadly follows this “collinearity rule” and is consistent with previously described Hox gene expression patterns (Fig. 2*A*) (13). In the central nervous system (CNS) of both larva and adult, the drivers likewise express in patterns that conform with those observed previously in the embryonic CNS (Fig. 2*B*) (33), or, when characterized, in the larval and adult CNS (19, 20, 22, 34–36). Apparent deviations were noted in some cases (Fig. 2*B*, white arrows), such as labeling with Antp^siTE^-Gal4, which typically spanned more than the three thoracic segments in which Antp expression is most prominent. However, Antp immunoreactivity in the early embryonic CNS has been reported to extend from the labial neuromere of the subesophageal ganglia to the seventh abdominal segment before subsequently intensifying in T1–T3 (33, 37, 38). This broader pattern is more consonant with the expression of the Antp^siTE^-Gal4 driver. To directly determine whether the driver was correctly reporting Antp expression, we double-labeled the adult CNS of Antp^siTE^-Gal4>UAS-nucLacZ flies with an anti-Antp antibody, which confirmed positive, albeit low-level immunostaining in regions robustly labeled by the Antp^siTE^-Gal4 driver (*SI Appendix*, Fig. S5*A*).

### Hox Gene Expression in Glia Revealed by Hox-Specific siTrojan Drivers.

Other unexpected labeling observed with the nuclear LacZ reporter included labeling of nuclei in nerves (Fig. 2*B*, yellow arrowheads). This was distinct from the uniform nerve staining (gray arrowheads) seen in some preparations, which was Gal4- and UAS-nucLacZ-independent and appeared to be an artifact of the anti-beta-galactosidase antibody used for staining (*SI Appendix*, Fig. S6 *A* and *B*). Nerves containing labeled nuclei were within the expected Hox expression domain except for the lab^siTE^-Gal4 driver, which together with the Ubx^siTE^-Gal4 driver also exhibited anomalous labeling (white arrows) in the brain lobes. The distribution and size of the labeled cells in the nerves and brain lobes, suggested that they were glia. Interestingly, Ubx gene transcription, without corresponding Ubx protein expression, has been reported in thoracic glia of late-stage embryos (39). We confirmed expression of both *lab* and *Ubx* in glia using repo-Gal80 to selectively repress Gal4 activity in those cells. In the presence of repo-Gal80, lab^siTE^-Gal4 labeling was largely restricted to neurons of the tritocerebrum in the CNS (Fig. 2*C*), consistent with previous reports (33). Similarly, repo-Gal80 suppressed Ubx^siTE^-Gal4 labeling in the abdominal nerves (Fig. 2*D*, arrowhead). Interestingly, double-labeling with anti-Ubx antibodies confirmed the presence of Ubx immunoreactivity in these nerve-associated glia, albeit at very low levels (Fig. 2*E*).

We observed a strong correlation between Ubx^siTE^-Gal4-driven reporter expression and immunoreactivity, with the driver labeling 97% of Ubx-immunopositive glia in the nerves (n = 703 cells). Only 1% of cells expressing reporter were immunonegative, indicating that the Ubx^siTE^-Gal4 driver coexpresses with the *Ubx* gene with high fidelity in these cells. Overall, close correspondence of anti-Ubx immunoreactivity and Ubx^siTE^-Gal4 expression was also observed in the neurons of the VNC. However, the anomalously labeled cells in the larval brain lobes were an exception. These cells were not immunostained by anti-Ubx antibodies and proved not to be glia. They appear to represent ectopically expressing cells in the Ubx^siTE^-Gal4 pattern.

### Fidelity of Expression of the Hox-Specific siTrojan Gal4 Drivers.

We also assessed driver fidelity in the CNS at the larval stage, where overlap in expression was not always as obvious as at the adult stage when expression was relatively stable. This was due to widely differing levels of anti-Hox immunostaining and Gal4-driven reporter expression, which led to large disparities in the two signals. While these disparities were generally less obvious in the segmental patterns of larval CNS labeling (*SI Appendix*, Fig. S5 *B* and *C*), they could be quite striking at the cellular level and some cells were labeled exclusively by either antibody or the reporter.

To assess the overall fidelity of expression in a way that allowed comparison across preparations, we examined the overlap of anti-Hox immunoreactivity and Hox^siTE^-Gal4-driven UAS-dsRFP reporter expression in a subset of identified motor neurons that express the transcription factor Even-skipped (Eve) (*SI Appendix*, Fig. S5 *C* and *D*). These neurons, known as Eve^+^ “U” motor neurons, are found in all segments of the VNC, but they express different Hox genes depending upon their position along the A-P axis (17, 40). Using anti-Eve immunostaining and antibodies against Antp, Ubx, and Abd-B, we directly compared Hox gene expression with the patterns of Gal4-driven reporter expression in the Eve^+^ U motor neurons. Correspondence between driver expression and immunoreactivity ranged from 79 to 89% (*SI Appendix*, Fig. S5*E*). False positive rates (i.e., reporter expression in immunonegative cells) for the three drivers averaged 9.0%. We conclude that despite differences in the apparent levels of Hox protein and Gal4 activity in individual cells, the driver fidelity is relatively high and that the siTrojan exon method provides a reliable means of generating gene-specific Gal4 lines.

### Intersectional Targeting of Motor Neurons along the A-P Axis Using siTrojan Split Gal4 Hemidrivers.

**Fig. 3.**
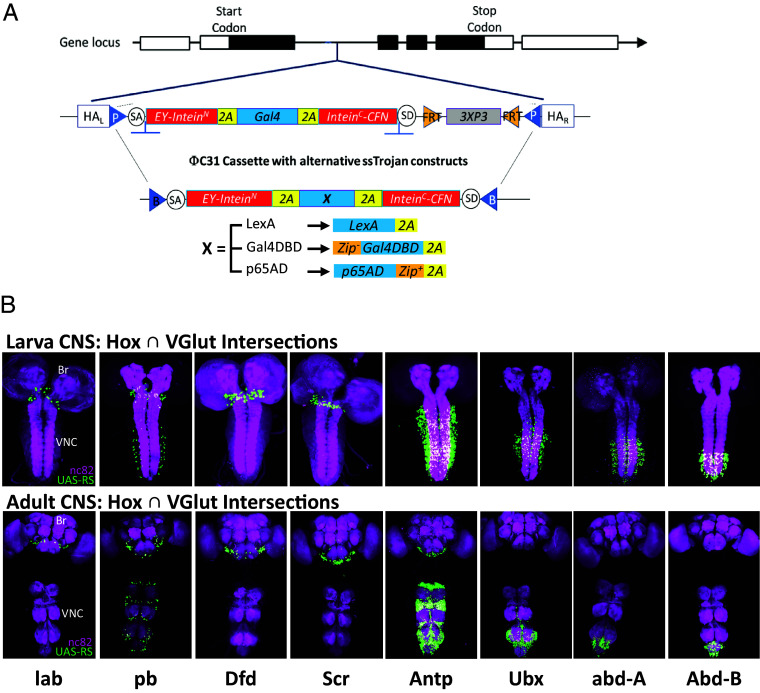
Targeting glutamatergic subsets of Hox gene–expressing neurons using siTrojan Split Gal4 cassettes. (*A*) siTrojan technology retains the modularity of the original Trojan exon system: An siTrojan-Gal4 insertion can easily be substituted with Split Gal4 hemidrivers, LexA::GAD drivers, or any other desired construct by ΦC31 integrase-mediated cassette exchange. (*B*) CNS expression of the Hox-specific hemidrivers in glutamatergic neurons of third instar larvae (*Top*) and adults (*Bottom*). For all Hox genes except *Dfd*, a p65AD hemidriver was used; for *Dfd* a Gal4DBD hemidriver was used. Br, brain; VNC, ventral nerve cord. Green, UAS-RS (RedStinger); magenta, nc82 neuropil labeling.

Like the original Trojan exons, siTrojans are modular. Flanking attP sites ensure that after insertion into the genome, any siTrojan Gal4 can be replaced by an alternative construct using ΦC31-mediated cassette exchange (Fig. 3*A*). To facilitate use of the siTrojan technology, we made attB-flanked constructs in all three reading frames that encode the Split Gal4 components Gal4DBD and p65AD, and the transcriptional activator, LexA::GAD. We tested the Split Gal4 constructs by converting all of the Hox Gal4 driver lines into either p65AD or Gal4DBD hemidrivers and combined these with complementary hemidrivers expressing the *VGlut* gene (Fig. 3*B*). *VGlut* encodes the vesicular glutamate transporter, which is expressed in *Drosophila* motor neurons. The Hox∩VGlut intersections thus target distinct complements of motor neurons along the A-P axis. Importantly, both siTrojan Gal4DBD and p65AD hemidrivers functioned well despite the T2A peptide fusions to their C termini (Fig. 3*A*).

**Fig. 4.**
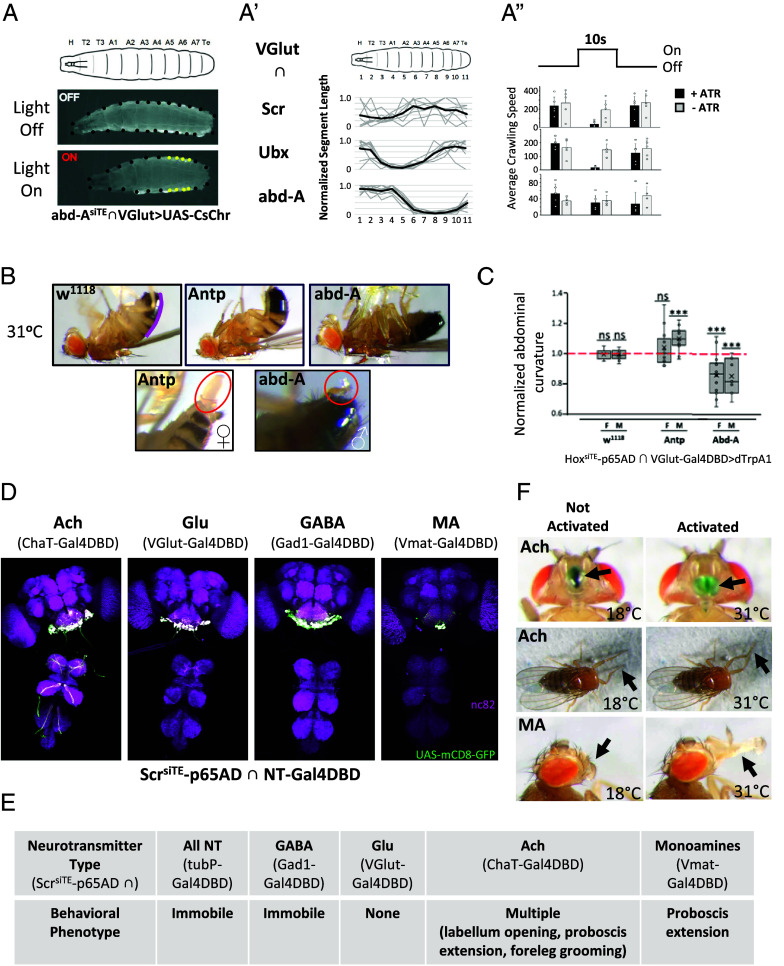
Activating subsets of Hox-gene expressing neurons has diverse behavioral effects. (*A*–*A*”) Activation of glutamatergic motor neurons in third instar larvae resulted in selective contraction of body wall segments, consistent with the pattern of Hox gene expression. (*A*) Stimulation of motor neurons in the *abd-A* expression pattern using the light-activated channel UAS-Cs.Chrimson.mVenus results in contraction of posterior segments when light is ON (*Bottom*, yellow dots) compared to when lights are OFF (*Middle*). In larval body wall schematic (*Top*): H, head; T2-T3, thoracic segments; A1-A7, abdominal segments; Te, fused terminal abdominal segments. (*A*’) Length changes in individual body wall segments (relative to lights off) during motor neuron activation for five animals of each indicated genotype/Hox gene. Gray values, segment lengths for each animal; dark lines, mean segment lengths. (*A*”) Crawling speed typically decreased during motor neuron activation (i.e., during 10 s lights-on stimulus vs. 10 s prior to or after the stimulus) in experimental animals of the same three genotypes indicated in (*A*’) that were fed all-trans-retinal (+ATR), a cofactor for Cs.Chrimson. Control animals of the same genotypes, but not fed ATR (-ATR), showed little change in locomotion with light. (*B*) In adult animals, dTrpA1-mediated stimulation of glutamatergic motor neurons expressing the Hox genes *Antp* and *abd-A* resulted in diverse behaviors that were sexually dimorphic. In males, these included persistent abdominal flexion (*Top* *Middle*, Antp) or extension (*Top Right*, abd-A) relative to the abdominal posture of w^1118^ control animals (*Top Left*) at the restricted temperature of 31 °C. Stimulation of abd-A-expressing motor neurons in males also robustly induced extension of the aedeagus and secretion of seminal fluid (*Bottom Right*, oval), while stimulation of Antp-expressing motor neurons in females induced egg extrusion in 5/6 animals (*Bottom Left*, oval). (*C*) Abdominal curvature was measured as described in *Materials and Methods* from the contours of the abdomen from segments A2 or A3 to the tip [see the magenta line for the w^1118^ animal in (*B*), *Top Left*]. Normalized abdominal curvature is the ratio of the curvature measured during motor neuron stimulation by dTrpA1 to the curvature measured prior to stimulation and is shown for both males and females of the indicated genotypes. N ≥ 10 for all conditions, significance determined by the *t* test. The dashed red line indicates no change in curvature with stimulation. (*D*) CNS expression pattern of the *Scr* gene subdivided according to the type of neurotransmitter used by component neurons. Four major subtypes were isolated by intersectional labeling using the Scr^siTE^-p65AD hemidriver and Gal4DBD hemidrivers with traditional Trojan exon insertions into the indicated genes. Green, UAS-mCD8-GFP; magenta, nc82 neuropil staining. (*E*) The behavioral phenotypes resulting from dTrpA1-mediated stimulation of either all Scr-expressing neurons, or those subsets shown in (*D*) that use a particular neurotransmitter. (*F*) Behavioral changes observed upon activation of cholinergic (Ach) and monoaminergic (MA) subsets of Scr-expressing neurons. *Left* and *Right* panels show individual flies at 18 °C and 31 °C, i.e., prior to and during dTrpA1-mediated stimulation, respectively. *Top* panels, dye placed on the labellum of a fly emphasizes its robust opening (arrows) upon stimulation of the cholinergic subset of *Scr*-expressing neurons. *Middle* panels, headless flies groom their forelegs (arrows) after the onset of stimulation of the same neuronal subset. *Bottom* panels, extension of the proboscis (arrows) accompanies activation of the monoaminergic subset of Scr-expressing neurons.

Motor neurons with cell bodies located in a specific segment of the larval VNC typically innervate muscles in the corresponding segment of the body wall. We therefore selected hemidrivers for the *Scr*, *Ubx*, and *abd-A* genes to span multiple distinct segments along the body wall and used the intersections of these hemidrivers with VGlut-Gal4DBD to optogenetically activate motor neurons using UAS-Cs.Chrimson.mVenus. In all cases, we observed a profound effect of motor neuron activation in response to a 10 s light pulse. During the pulse, the body wall segments controlled by the affected Hox-expressing motor neurons contracted constitutively (Fig. 4 *A* and *A’*), with contraction resulting in head-lifting for the Scr∩VGlut animals (Movie S1). Activation of motor neurons in the Scr and Ubx patterns substantially blocked forward peristaltic locomotion (Fig. 4*A”*).

At the adult stage, activation of neurons within the Hox∩VGlut intersections similarly resulted in specific behavioral effects. These effects were more varied than those seen in the larva, reflecting the greater complexity and sexual dimorphism of the adult neuromuscular system. In addition, activation of inhibitory glutamatergic interneurons may have influenced behavioral phenotypes. Stimulation of neurons in the Antp and abd-A patterns using the thermogenetic dTrpA1 channel differentially drove abdominal bending: Antp∩VGlut neuron activation promoted abdominal flexion, particularly in males, while abd-A∩VGlut neuron activation promoted abdominal extension in both sexes (Fig. 4 *B*, *Top*; Fig. 4*C*). This might occur if Antp motor neurons control ventral muscles of the abdomen, while abd-A motor neurons regulate dorsal muscles, a hypothesis that remains to be tested. Interestingly, activation of both sets of neurons consistently induced motor effects in the reproductive organs. Activation of the Antp motor neurons typically caused extrusion of an egg in females (n = 5/6; Fig. 4 *B*, *Bottom Left*), while activation of abd-A motor neurons caused extension of the aedeagus in males and secretion of seminal fluid (Fig. 4 *B*, *Bottom Right*). A population of abdominal motor neurons expressing Insulin-like peptide 7 have been previously implicated in egg extrusion (41), and glutamatergic neurons that express the transcription factor Doublesex have been previously implicated in seminal fluid transfer in males (42). It will be interesting in future investigations to see whether these populations lie within the expression patterns of the *Antp* and *abd-A* genes, respectively.

### Hox Gene siTrojan Hemidrivers Facilitate Behavioral Circuit-Mapping.

These examples illustrate how Hox gene–specific hemidrivers can be used to subdivide glutamatergic neurons into distinct subsets and probe their contributions to behavior. Similar neuronal subdivisions can be performed for any gene of interest for which a hemidriver is available, and conversely, diverse hemidrivers can be used to subdivide any given Hox gene expression pattern to isolate neurons involved in developmental, physiological, or behavioral processes of interest. To illustrate this application and its potential utility in mapping behavioral circuits, we subdivided *Scr*-expressing neurons according to their neurotransmitter usage and tested the behavioral responses to thermogenetic activation with UAS-dTrpA1. As before, we used VGlut-Gal4DBD with Scr-p65AD to isolate glutamatergic neurons and additional Gal4DBD hemidrivers for the genes *ChaT*, *Gad1*, and *Vmat*, to isolate cholinergic, GABAergic, and monoaminergic neurons, respectively. The subsets of neurons isolated using these intersections are shown in Fig. 4*D* and the behavioral effects of activation are listed in Fig. 4*E*. Interestingly, activation of the entire subset of *Scr*-expressing neurons (using the ubiquitously expressed hemidriver tubP-Gal4DBD) resulted in behavioral immobility. This phenotype evidently derived from activation of the GABAergic subset of neurons since the same phenotype was observed when these neurons alone were activated. Glutamatergic neuron activation surprisingly had no phenotype, but robust behavioral responses were seen upon activation of both the cholinergic and monoaminergic subsets of neurons. The response to activation of cholinergic neurons was most complex. Flies constitutively opened the labellum of the proboscis upon stimulation (Fig. 4 *F*, *Top*) and typically initiated foreleg grooming shortly thereafter (Fig. 4 *F*, *Middle*; Movie S2). In fixed flies, this phenotype was observed only when the animals were decapitated, but it was readily induced in intact, freely moving animals. These results suggest the activation of multiple neuronal circuits, consistent with the fact that Ach is the major excitatory neurotransmitter of the fly CNS. Activation of the small subset of Vmat-expressing neurons within the *Scr* expression pattern drove robust proboscis extension (Fig. 4 *F*, *Bottom*). This is consistent with *Scr*’s expression in the labial neuromere of the SEZ and illustrates the kind of anatomical resolution that can be leveraged in circuit-mapping studies using the Hox-specific reagents introduced here.

## Discussion

The siTrojan technique introduced here permits genetic targeting of cells based on their expression of a native gene of interest. By integrating split intein technology, the siTrojan technique mitigates the mutagenic effects of the previously introduced Trojan exon method while retaining its modularity. To demonstrate the efficacy of the modified method, we have generated a comprehensive panel of gene-specific siTrojan Gal4 drivers and Split Gal4 hemidrivers for the Hox transcription factors. We demonstrate the use of this Hox-specific toolkit in targeting neuronal types according to their positions along the neuraxis, an application that will facilitate mapping neural circuits. The generality and versatility of the siTrojan exon method should make it a valuable tool for targeting cells expressing not only transcription factors, but other genes that have hitherto resisted creation by other methods.

### Advantages and Limitations of siTrojans: Modularity and Reduced Mutagenicity.

A key factor in the success of the Trojan exon technology—introduced by Diao et al. (4) and Gnerer et al. (5) and subsequently modified by Nagarkar-Jaiswal (1) —derives from its modularity: In a single-step, ΦC31-mediated cassette exchange can be used to replace any Trojan exon or Trojan-like cassette (e.g., MiMIC or CRIMIC) with another Trojan exon. This can, in fact, be easily achieved via straightforward genetic crosses when exchanging in either Gal4 or Split Gal4 Trojan exons (4, 23). This simple modularity has additionally permitted the ready generation of lines with LexA and Gal80 expression from Trojan Gal4 lines and is not readily possible using other common techniques for making gene-specific driver lines. The siTrojan exon method introduced here retains this essential advantage.

Importantly, the siTrojan technique also reduces the mutagenicity of the original method. It does not, however, completely eliminate it. While viable heterozygotes could be generated by siTrojan insertions into sites resistant to transformation by classical Trojan exons, homozygous animals typically exhibited impaired function. In fact, siTrojan insertions into all Hox genes except *pb* were homozygous lethal. Lethality may result from residual dominant negative effects of incompletely spliced translation products, since our experiments with ChaT indicate that native protein reconstitution is only partial. Alternatively, homozygous lethality may result from impaired Hox protein function due to the short peptide insertions introduced by the siTrojan method. In general, however, our success in making a nearly full palette of Hox gene–specific reagents confirms the feasibility of the approach. Only for the *Scr* gene, which has a single coding intron, were we unable to make a Gal4 driver, and for this gene we were able to directly generate a siTrojan Split Gal4 p65AD hemidriver. The p65AD strategy has previously proved successful with classical Trojan exon insertions for which Gal4 insertions are problematic, as for the *amon* and *Gad1* genes described by Diao et al. (4).

### Split Intein Technology in Reconstituting Arbitrary Native Proteins.

The split intein technology on which siTrojans are based has been used in a variety of biological contexts to reconstitute the function of split proteins from nonfunctional components (8, 10). In *Drosophila*, split inteins have been used in expression systems that rely on the reconstitution of split Cre, split Gal4, and split GeneSwitch molecules (24, 43). In all of these cases, the split protein has been carefully engineered to permit scarless excision of the split intein to leave the protein in its native form after ligation. However, scarless excision is generally difficult to ensure in arbitrary protein contexts because self-splicing efficacy depends on the sequences flanking the split inteins, a phenomenon known as “extein dependence” (26).

In an effort to limit the extein dependence of our siTrojan constructs, we flanked the Cfa^C^ and Cfa^N^ by amino acid residues known to promote ultrafast trans-splicing. This necessarily increases the size of the peptide insertions of our siTrojans into the targeted proteins by five residues (i.e., EYCFN), which may increase the severity of the introduced mutation. It is possible that smaller insertions could be achieved with alternate construct designs. Mutagenesis experiments on Npu, the split intein from which Cfa is derived, indicate that substituting other amino acids for the E and N residues is often—though not always—well-tolerated (44). A recently engineered variant of Cfa with reduced Cfa^C^ extein-dependence also suggests the possibility of reducing the size of peptide insertions (26). Until a split intein is developed that is capable of scarless trans-splicing in all protein environments, however, the trade-off between split intein efficacy and size of the mutational insertion will remain. In the meantime, the success of the approach taken here indicates that extein dependence does not limit use of split inteins to applications in which the protein context is defined. Our results indicate that siTrojan constructs support split intein activity when inserted into a wide range of protein contexts and similar in vivo applications of this technology in *Drosophila* and other animals should be possible.

### The Hox Gene–Specific Driver Toolkit.

Hox genes have been a source of intense interest since their discovery and have recently enjoyed renewed attention due to their role in the development of motor circuits for behavior (17, 45). Movements involved in feeding, locomotion, and reproduction require appendages found at different positions across the A-P axis and evidence suggests that establishing neuromuscular connectivity for controlling these movements depends on the actions of Hox genes expressed in different segments (15, 16). In addition, the serially ordered expression of Hox genes along the A-P axis of the nervous system represents a potential resource for dissecting motor circuit elements located at different levels of the neuraxis using intersectional methods (20). For all of these applications, tools for targeting Hox gene–expressing cells are important.

Recent efforts to make Hox gene–specific drivers have focused on “knock-in” approaches that replace the entire coding sequence of a Hox gene with a transcriptional activator (20), or in-frame fusion approaches (46) that insert a Gal4-T2A module at the position of the start codon (19). Using these methods, various LexA, Gal4, and Split Gal4 lines have been generated for the *Dfd*, *Scr*, *pb*, and *Antp* genes (*SI Appendix*, Table S1). Where characterized, the expression patterns of these lines in the larval and adult CNS are similar to those reported here, and although fidelity of expression has not been directly interrogated, evidence suggests that it is reasonably high. For example, the *Dfd*- and Scr-LexA lines express in the expected neuromeres of the SEZ and the numbers of neurons labeled conform well with estimates obtained by other methods (47).

Our own results indicate a generally strong correlation between anti-Hox immunostaining and siTrojan Hox-Gal4 driven reporter expression, especially in the adult CNS. An as yet unexplained exception is the anomalous labeling of sparsely distributed cells in the brain lobes by the Ubx^siTE^-Gal4 driver, which may result from the introduction of a novel enhancer (active in the ectopically expressing cells) via siTrojan-Gal4 insertion. The regional specificity of the Hox Gal4 drivers is also good in the larval CNS, but we note some disparities between reporter expression and immunolabeling in individual cells. While these discrepancies may result from driver infidelity, alternative explanations are possible. Unusually divergent levels of reporter expression and immunolabeling suggest that the kinetics of gene expression or protein perdurance may differ between the UAS-reporters and Hox genes. UAS-reporter expression depends on maturation of, and transcriptional activation by, Gal4 and will therefore lag expression of the Hox gene whose activity it reports on. This could produce Hox immunopositive cells with weak, or no, reporter labeling. Conversely, perdurance of Hox proteins after the termination of Hox gene transcription and degradation of the reporter could produce immunopositive cells with little or no corresponding reporter signal. Consistent with these possibilities, Hox gene regulation is known to be dynamic, complex, and cell type dependent in both flies and mice (48, 49). The siTrojan drivers presented here provide an additional tool for studying this regulation, and coupled with more detailed tracking of Hox protein expression and degradation they may help identify the source of the developmental disparities in expression we observe.

In any case, the segmentally selective expression patterns of our Split Gal4 Hox gene lines will make them useful in the dissection of neural circuits as indicated by the applications shown here. Current circuit-mapping efforts in *Drosophila* rely heavily on intersectional strategies using Split Gal4 in which specific neurons are targeted based on their expression of genes with largely distinct, but overlapping, anatomical expression (21). Our reagents significantly expand the number of available Hox Split Gal4 hemidrivers, which are currently limited to *Dfd*, *Ubx,* and *Abdominal-B* (*SI Appendix*, Table S1). Moreover, the modularity of the siTrojan method will allow lines expressing other factors useful for intersectional targeting, such as LexA, to be easily generated on demand.

The ability to easily generate multiple lines expressing different effectors in the same gene-specific pattern is not found in the Hox-specific drivers created by other means, and as noted above is a unique feature of the Trojan and siTrojan method. Kanca et al. (50) have estimated that roughly half of the protein-coding genes in *Drosophila* contain introns that are common to all isoforms and thus suitable for the introduction of synthetic exons, which indicates that the method should be broadly useful. To facilitate use of the method, we introduce here attB-flanked cassettes for ΦC31-mediated exchange of siTrojan Gal4, Gal4DBD, p65AD, and LexA::GAD constructs, in addition to attP-flanked siTrojan Gal4 cassettes for CRISPR/Cas9-mediated insertion into targeted introns. Together, these reagents provide a powerful set of tools for generating gene-specific drivers in *Drosophila* and, like the Hox-specific toolkit described, should be broadly useful to researchers studying developmental and neurobiological processes in the fly.

## Materials and Methods

Full methods are available in *SI Appendix*, *Extended Methods*.

### Fly Lines.

Strains previously described by Diao et al. (4) include tubP-Gal4DBD, VGlut^MI04979^-Gal4DBD, VGlut^MI04979^–p65AD, and ChaT^MI04508^–Gal4DBD. UAS-6XmCherry, Gad1^MI09277^–Gal4DBD, UAS-Cs.Chrimson.mVenus, and UAS-nucLacZ were from the Bloomington Drosophila Stock Center. Repo-Gal80 and dUAS-dTrpA1 were gifts from Tzumin Lee and Paul Garrity, respectively. Transgenic flies created in this study are described in *SI Appendix*.

### Molecular Biology.

Constructs for in vitro testing of the Cfa split intein were made by cloning the LexA-Cfa^N^ and Cfa^C^-p65AD fusions into the pPacPL-PL-mCD8-D2A-Gal4 plasmid described by Diao and White (46). Coding sequences of Cfa^N^ and Cfa^C^ were derived by reverse translation from protein sequences in Stevens et al. (12). The resulting DNA sequences were *Drosophila* codon-optimized. Nucleotides encoding amino acids EY and CFN were added to the 5′ and 3′ ends of the Cfa^N^ and Cfa^C^ sequences, respectively. The LexA and nlsp65AD sequences were from Diao et al. (4). siTrojan exon constructs were modified from the Trojan exon constructs of Diao et al. (4) using pTGEM(0) as a backbone. The guide RNAs (gRNAs) used to generate the Hox gene Gal4 drivers and Scr^siTE^-p65AD hemidriver by Crispr/Cas9 are listed in the Extended Methods and were ligated into the *Bbs* I-digested pBS-U6A1-sgRNA-short vector of Rem et al. (51). For the siTrojan constructs, 100 to 500 bp of genomic sequence flanking the gRNA sequences served as left and right homologous arms. The plasmids “d2EGFP” and pDsRed-Express-DR were used to amplify dsGFP and dsRFP, which were cloned into the pUASTattB vector (Addgene).

### Transgenic Fly Lines.

For lines made using Crispr/Cas9, plasmid DNA for each siTrojan-exon construct was microinjected into embryos of {nos-Cas9} attP40 flies (51) together with sgRNA. Hox gene hemidriver lines and non-Hox gene drivers and hemidrivers were made by ΦC31-mediated cassette exchange.

### S2 Cell Culture.

S2 cells were grown to a density of 10^6^ cells mL^−1^ and transfected with 1.0 µg of each DNA construct. pPac-PL-nlsLexA-Myc-EYCfaN-T2A-3XHA, pPac-PL-3XHA-T2A-CfaC-CFN-nlsp65AD and 13X LexAop2-6XmCherry plasmid DNAs were cotransfected and assayed for LexA-p65AD activity by mCherry expression. As negative controls, plasmid DNAs for each individual split intein construct were cotransfected with 13X LexAop2-6XmCherry. Cells were analyzed for fluorescence and counted after 3 d incubation at 25 °C.

### Immunoblotting and Immunohistochemistry.

The following primary antibodies were used at the indicated dilutions for immunolabeling: Rabbit anti-β-galactosidase (Bio-Rad; 1:100), rabbit anti-Eve (GenScript; 1:1,000), chicken anti-mCherry (Novus; 1:1,000); chicken anti-GFP (Aves; 1:1,000). Mouse anti-Ubx (1:200), anti-Abd-B (1:400), anti-Antp (1:400), anti-nc82 (1:30), anti-ChaT (1:100), and anti-elav (1:100) were all obtained from the Developmental Studies Hybridoma Bank (University of Iowa).

#### Embryo preparations.

Fly embryos were collected, dechorionated, and fixed according to standard procedures (*SI Appendix*, *Extended Methods*) before being mounted on glass coverslips and imaged by confocal microscopy using both fluorescence and transmitted light.

#### CNS preparations.

Adult and larval nervous systems were dissected and stained according to standard procedures.

#### Analysis of Eve^+^ U motor neuron immunostaining.

CNS preparations expressing UAS-dsRFP or UAS-dsGFP under the control of Antp^siTE^-, Ubx^siTE^-, or Abd-B^siTE^-Gal4 were immunostained with anti-Eve, anti-Hox, and anti-mCherry (for dsRFP) or anti-GFP (for dsGFP) antibodies and analyzed. For each of three preparations for each Hox^siTE^-Gal4 driver, 3 to 5 Eve^+^ cells (U motor neurons) were identified in five to six VNC hemisegments in which the relevant Hox gene was expressed and scored for coexpression of anti-GFP/mCherry or anti-Hox. Eve^+^ cells that were both Hox^+^ and GFP/RFP^+^ were counted and “Driver Fidelity” was determined by dividing the sum of the cells counted in all three preparations by the total number of Eve^+^ cells examined (multiplied by 100). Mismatched labeling was assessed by similarly calculating the percentage of Hox^+^ GFP/RFP^-^ (“false negatives”) and GFP/RFP^+^ Hox^-^ (“false positives”) cells.

### Behavioral Assays.

#### Larval behavior analysis.

Larval behavior was analyzed as described previously (52) and images were processed in Fiji (53). To measure speed of locomotion, the change in position of the larva’s posterior tip was calculated as distance (µm) over time. To measure segment lengths, the multipoint selection tool on ImageJ was used to distinguish segment boundaries and establish regions as shown in Fig. 4*A’*.

#### Adult behavioral analysis.

Flies were glued by the thorax to a 10 µL pipette tip and positioned above a Peltier plate. Plate temperatures were adjusted to yield final temperatures at the fly of 22 °C (inactive condition) or 31 °C (stimulation condition) and behavior was monitored by videorecording. Abdominal curvature was measured using the Kappa-Curvature Analysis tool in Fiji (53) from the second or third abdominal segment to the posterior tip. Mean curvature for each condition was calculated by averaging six measurements spaced over 30 s intervals either before or after stimulation.

## Supplementary Material

Appendix 01 (PDF)

Movie S1. **Head lifting in response to activation of Scr^siTE^∩VGlut neurons.** Speed: 3X.

Movie S2. **Foreleg grooming by a decapitated fly in response to activation of Scr^siTE^∩ChaT neurons.** Speed: 3X.

## Data Availability

Primary numerical Data and Software data have been deposited in figshare (https://doi.org/10.6084/m9.figshare.c.7018752) (54).
