# Supplementary material for: Hox gene–specific cellular targeting using split intein Trojan exons
Source: Proc Natl Acad Sci U S A. 2024 Apr 11;121(17):e2317083121. doi: 10.1073/pnas.2317083121 (PMC11047080; doi:10.1073/pnas.2317083121)
Supplement: Supplementary file 1 — Appendix 01 (PDF) [file pnas.2317083121.sapp.pdf]

PNAS

Supporting Information for

Hox Gene Specific Cellular Targeting Using Split intein Trojan Exons

Fengqiu Diao[1], Deeptha Vasudevan[2], Ellie S. Heckscher[2], and Benjamin H. White[1*]

Corresponding author: Benjamin White
E-mail: benjaminwhite@mail.nih.gov

This PDF file includes:

Extended Methods
Figures S1 to S6
Tables S1 to S2
Legends for Movies S1 to S2
SI References

Other supporting materials for this manuscript include the following:

Movies S1 to S2

Extended Methods

Fly lines:

Drosophila melanogaster stocks were maintained with cornmeal-molasses-yeast medium and housed at 25°C in 65% humidity. Strains previously described by Diao et al. (1) used in this study include: tubP-Gal4DBD, VGlut^{MI04979}-Gal4DBD, VGlut^{MI04979}–p65AD and ChaT^{MI04508}–Gal4DBD. The UAS-6XmCherry (BL52268), Gad1^{MI09277}–Gal4DBD (BL82987), UAS-Cs.Chrimson.mVenus (BL55135), and UAS-nucLacZ (BL3956) were from the Bloomington Drosophila Stock Center. The repo-Gal80 and dUAS-dTrpA1 lines were kind gifts from Tzumin Lee and Paul Garrity, respectively. Transgenic flies created in this study include: lab^{siTE}-Gal4, lab^{siTE}-p65AD, pb^{siTE}-Gal4, pb^{siTE}-p65AD, Dfd^{TE}-Gal4, Dfd^{siTE}-Gal4^{1}, Dfd^{siTE}-Gal4^{2}, Dfd^{siTE}-Gal4DBD, Scr^{siTE}-p65AD, Antp^{siTE}-Gal4, Antp^{siTE}-p65AD, Ubx^{siTE}-Gal4, Ubx^{siTE}-p65AD, abd-A^{siTE}-Gal4, abd-A^{siTE}-p65AD, Abd-B^{siTE}-Gal4, Abd-B^{siTE}-p65AD, ChaT^{siTE}-Gal4, and crc^{siTE}-Gal4 in addition to the Vmat-Gal4DBD line and the destabilized fluorescent reporter lines UAS-dsGFP and UAS-dsRFP. All plasmid injections to generate transgenic lines were performed by Rainbow Transgenic Flies, Inc. (Camarillo, CA)

Molecular Biology:

Primers for PCR and gBloc DNA fragments for cloning were synthesized by Integrated DNA Technologies, Inc. (Coralville, Iowa). Restriction enzymes, T4 DNA ligase, and PCR amplification kit (Q5 Hot Start High-Fidelity 2X Master Mix) were from New England Biolabs, Inc. (Ipswich, MA). Plasmid DNA was isolated using ZymoPURE Plasmid Miniprep and Midiprep Kits from Zymo Research Corporation (Irvine, CA) and DNA sequencing was performed by Psomagen USA (Rockville, MD). Unless otherwise

noted, cloning reactions and bacterial transformations used In-Fusion 2X mix and Stellar Gold Competent Cells from Takara Bio USA, Inc. (San Jose, CA).

Destabilized EGFP (dsGFP) and DsRed (dsRFP) constructs: The plasmids "d2EGFP" (a kind gift from Dr. Gyeong-Hun Baeg of Macau University of Science and Technology) and pDsRed-Express-DR (Takara Bio USA, Inc.) were used to amplify dsGFP and dsRFP using forward and reverse primers that included 15 bp of sequence homologous to the pUASTattB vector (Addgene). The PCR products were cloned into pUASTattB (digested with *EcoR* I/*Not* I) using the In-Fusion Cloning Kit (Takara Bio UAS, Inc.) to get pUASTattB-dsGFP and pUASTattB-dsRFP. These constructs were used to introduce UAS-dsGFP and UAS-dsRFP into the attP40 landing site on the Drosophila 2nd chromosome by ΦC31-mediated recombination.

Constructs pPac-PL-LexA-Myc-EY-Cfa^{N}-T2A-3XHA and pPac-PL-HA-T2A-Cfa^{C}-CFN-nlsp65AD for in vitro testing in S2 Cells: To generate coding sequences of the Cfa^{N} and Cfa^{C} split inteins, the corresponding protein sequences from Stevens et al. (2) were reverse translated and the resulting DNA sequences codon-optimized for *Drosophila*. To ensure efficient splicing, nucleotides encoding amino acids residues Glu-Tyr and Cys-Phe-Asn were added to the 5' and 3' ends of the Cfa^{N} and Cfa^{C} sequences, respectively. The LexA and nlsp65AD sequences were from Diao et al (1). To make the pPac-PL-LexA-Myc-EY-Cfa^{N}-T2A-3XHA and pPac-PL-HA-T2A-Cfa^{C}-CFN-nlsp65AD constructs, pPacPL-PL-mCD8-D2A-Gal4 plasmid DNA from Diao and White (3) was first digested with BamH *I*, Not *I* and Kpn *I* and then mixed with the gBloc DNA fragment encoding either LexA-Myc-EY-Cfa^{N}-T2A-3XHA or HA-T2A-Cfa^{C}-CFN-

nlsp65AD in In-Fusion 2X Mix. The mixture was incubated for 15 min at 50°C and then transformed into competent cells.

siTrojan Gal4, p65AD, and Gal4DBD Constructs: The siTrojan constructs were modified from the original Trojan exon constructs described by Diao et al. (1) using the pTGEM(0) vector as a backbone. DNA sequences for the Cfa^N and Cfa^C split inteins were similar to those described above and included coding sequences for the flanking EY and CFN amino acids as described above. In addition, to accommodate insertion into all three possible reading frames extra nucleotides were added before the sequences encoding the EY and/or after CFN, as indicated in Fig. S1A. Briefly, DNA constructs in the form attP-SA-EYCfa^N-T2A-(Gal4/p65AD)-T2A-Cfa^CCFN-SD-FRT-3XP3-RFP-FRT-attP were synthesized by Integrated DNA Technologies, Inc. (Coralville, Iowa) for all three reading frames. (Constructs used to convert existing insertions substituted attB for attP and Gal4DBD for Gal4 or p65AD, when needed.) For some constructs, DNA insertion fragments could not be synthesized directly. In these cases, 2-3 short DNA fragments with overlapping ends were synthesized and combined by PCR. DNA constructs were cloned into the pTGEM(0) plasmid DNA digested with *Not* I and *Asc* I.

Hox-specific siTrojan exon drivers: The seven Hox gene Gal4 drivers and Scr^{siTE}-p65AD hemidriver were made using Crispr/Cas9-mediated homologous recombination. Guide RNAs (gRNAs) conforming to the sequences shown in the table below were selected to make pBS-U6A1-sgRNA constructs for each of the Hox genes. Preference was given to sequences in introns that did not interrupt protein domains or contain highly conserved DNA sequence. For *Dfd*, gRNAs were selected for two introns (i.e. introns 2 and 3) with reading frame phases of 1 and 0, respectively. For several other genes (*pb*, *abd-A* and

Abd-B), multiple gRNAs were selected when use of the initially selected gRNA(s) failed to generate transformants. Synthesized, complementary oligos were annealed to form each gRNA and ligated into the *Bbs* I-digested pBS-U6A1-sgRNA-short vector of Rem, et al. (4). Ligated plasmid DNA products were transformed into DH5α competent cells from Thermo Fisher Scientific (Waltham, MA). For the siTrojan constructs, DNA fragments representing 100-500 bp of the sequence flanking the gRNA sequences were synthesized to serve as left and right homologous arms. These arms were then cloned into siTrojan exon vectors digested with *Not* I (left arm) or *Asc* I (right arm).

**Hox Gene**	**gRNA sequence (Intron; driver)**
lab	GTTGAGAGTAATGCACTAAG GGG (1; lab^{siTE}-Gal4)
pb	TTCCAAATTTCCTATAAATT AGG (3)<br>GAGTCAGTCATCTATAGTGA TGG (7; pb^{siTE}-Gal4)
Dfd	TCCGTTGAGATTTGATAGG AGG (2; Dfd^{siTE}-Gal4^{1})<br>CTCGATTACGCATTCTCTCT TGG (3; Dfd^{siTE}-Gal4^{2})
Scr	GAACTTCCCGTCTAGTCTAT AGG (2; Scr^{siTE}-p65AD)
Antp	TGGTTGGCGAAATATGCGGG AGG (3; Antp^{siTE}-Gal4)
Ubx	GAAAAGACAAACACCTTAT TGG (1; Ubx^{siTE}-Gal4)
abd-A	ACCTTTAAGAAGATGTTTCC GGG (3)<br>GCAAGTTAGTTAAGAGTTTA AGG (6; abd-A^{siTE}-Gal4)
Abd-B	GAAGTTGCTTACAACTGATG TGG (4; Abd-B^{siTE}-Gal4)<br>ATAGTAAAGACTCGTTATTA TGG (5)<br>TGAATTCAGTTCAAGTCAGT AGG (6)

***Transgenic Fly Lines*:**

For lines made using Crispr/Cas9-mediated homologous recombination, plasmid DNA for each siTrojan exon construct was microinjected into embryos of {nos-Cas9} attP40 flies (4) together with sgRNA. Transformants were identified by crossing injected animals with w;+;Dr/TM3,Sb flies and screening for the presence of the 3XP3-RFP selection marker (i.e. red fluorescent eyes) in the progeny.

Hox gene hemidriver lines and the $ChaT^{siTE}$ and crc^{siTE} Gal4 driver lines were made by ΦC31-mediated conversion as follows. Midiprep plasmid DNAs containing the desired siTrojan exons in the correct reading frame were injected together with ΦC31 plasmid DNA into the embryos of flies with Hox gene siTrojan drivers made by Crispr/Cas9 or flies with MiMIC insertions (5). Transformants were isolated by crossing injected animals with w;;Dr/TM3,Sb flies (for siTrojan Gal4 transformants) or yw;;Dr/TM3,Sb flies (for MiMIC convertants) and screening for loss of the 3XP3-RFP selection marker or yellow selection marker, respectively. Transformants were crossed with w;;Dr/Tm3,Sb flies to make stable lines and their expression patterns were confirmed by crossing to w;UAS-2X EGFP flies (for Gal4 lines), yw;Tub-Gal4DBD;UAS-2X EGFP flies (for p65AD hemidrivers) or yw;Tub-VP16AD,UAS-2X EGFP flies (for Gal4DBD hemidrivers). The Vmat-Gal4DBD line was likewise made by ΦC31-mediated conversion of a Trojan exon Gal4 driver, $Vmat^{MI07680\text{-}TG4.0}$ available from the BDSC.

S2 Cell Culture:

S2 cells were grown in serum-free HyQ-CCM3 medium from VWR International (Radnor, PA) to a density of 10^6 cells ml^{-1} in 6-well plates. Cells in each well were transfected with 1.0 μg of each purified DNA construct using the FuGENE6 Transfection

Reagent from Roche Diagnostics USA (Indianapolis, IN). pPac-PL-nlsLexA-Myc-EYCfaN-T2A-3XHA, pPac-PL-3XHA-T2A-CfaC-CFN-nlsp65AD and 13X lexAop2-6XmCherry plasmid DNAs were co-transfected in experiments in which LexA-p65AD activity was monitored by 13X lexAop2- 6X mCherry expression. In two negative controls, pPac-PL-nlsLexA-Myc-EY-CfaN-T2A-3XHA or pPac-PL-3XHA-T2A-CfaC-CFN-nlsp65AD and 13X LexAop2-6XmCherry plasmid DNAs were co-transfected. Cells were analyzed for fluorescence using a Nikon C2 personal confocal microscope after 3d incubation at 25 °C. To quantify the density of mCherry-labeled cells in each of the three conditions (i.e. the two control conditions, with only one of the two split intein constructs, and the experimental condition with both split intein constructs), two wells containing cells transfected under each condition were photographed and a mask with the same six randomly chosen circular fields was overlaid on each photograph. The number of labeled cells was then counted in the 12 fields for each condition and cell counts were averaged.

***Immunoblotting and Immunohistochemistry*:**

Immunoblotting of ChaT: Adult CNS preparations were dissected in PBS and combined with 4 μl of 1X LDS loading buffer with 0.1M DTT. 8 μl samples (2 CNS equivalents) were boiled 5 min and then loaded on a 4-12% Bis-Tris NuPAGE gel. After electrophoresis in MES running buffer, proteins were transferred to a nitrocellulose membrane using Tris-Glycine transfer buffer (all reagents from ThermoFisher Scientific Inc.). The blot was incubated at room temperature overnight with anti-ChaT (4B1; 1:100) and anti-Elav (9F8A9; 1:100) antibodies from the Developmental Studies Hybridoma Bank and then for 2 h at room temperature with HRP conjugated goat anti-mouse

secondary antibody (1:1000; Jackson ImmunoResearch). The blot was imaged on a ChemiDoc Imaging System (BioRad Laboratories) in SuperSignal West Femto Maximum Sensitivity Substrate (ThermoFisher Scientific).

Immunohistochemistry: The following primary and secondary antibodies were used at the indicated dilutions for immunolabeling: Rabbit anti-β-galactosidase (Bio-Rad; 1:100), rabbit anti-Eve (GenScript; 1:1000), chicken anti-mCherry (Novus; 1:1000); chicken anti-GFP (Aves; 1:1000). Mouse anti-Ubx (1:200), anti-Abd-B (1:400), anti-Antp (1:400), and anti-nc82 (1:30) were all obtained from the Developmental Studies Hybridoma Bank (DSHB, Iowa University). Secondary antibodies were from ThermoFisher Scientific (AlexaFluor 488, 568 and 647; 1:500) or Jackson ImmunoResearch (DyLight 405, AlexafFluor 488 and 647, and Cy3; 1:400).

Embryo preparations: Fly embryos were collected on grape juice agar plates at room temperature for 1 hr and then transferred to 18°C for 20- 23 hr. Embryos were then dechorionated with 100% bleach, washed with water, and immersed in heptane (Millipore Sigma, St. Louis, MO) before transfer into glass vials with heptane. Embryos were fixed at room temperature for 20 min in 4% paraformaldehyde in PEM buffer (0.1M PIPES, 1mM $MgCl_2$, 1mM EDTA, pH 6.9)/heptane after vigorous shaking for 15s. After removal of fixation buffer in the lower phase, methanol was added and vials were again shaken vigorously for 15s. After removing the whole solution (including floating, damaged embryos), the embryos on the bottom were given a final methanol wash and mounted on glass coverslips. Z-series (1 μm step size) were collected through each embryo using both fluorescence and transmitted light. A maximum projection image of the mCherry fluorescence for each embryo was merged using Adobe Photoshop (Adobe,

Inc., San Jose, CA) with a cropped (two Z-steps) projection of the transmitted light image of the embryo structure.

CNS preparations: Adult nervous systems were dissected in phosphate buffered saline (PBS), fixed in 4% paraformaldehyde/PBS for 30 min and then incubated in 4% paraformaldehyde/PBS plus 0.5% Triton X-100 for 15 min. Samples were immunostained as described previously (6), mounted in VectaShield (Vector Laboratories) and imaged by confocal microscopy with *Z*-series collected in 1 μm increments on a Nikon A1R microscope. Unless otherwise noted, the images shown are maximum projections of the entire Z-stack. Larval nervous systems were dissected in Baines' solution and excised brains were adhered to poly-lysine-coated coverslips and fixed for 7 min in 4% paraformaldehyde solution as previously described (7). Brains were washed 3X with PBS containing 0.1% Triton X-100 (PBT), blocked 1 hr in PBT containing 2% normal donkey serum (NDS), and stained with primary antibody, in PBT at 4°C overnight. After washing, samples were stained with secondary antibody at room temperature for 1 hr, washed again, and applied with increasing percentages of ethanol in water (30, 50, 70, 95, 100) series to replace PBT. Samples were then immersed into xylene for clearance, and then mounted in DPX (Sigma, MI). Images were acquired using a Zeiss LSM 800 confocal microscope and were processed and analyzed using ImageJ.

Analysis of Eve^{+} U motor neuron immunostaining: CNS preparations from animals expressing either UAS-dsRFP or UAS-dsGFP under the control of Antp^{siTE}-, Ubx^{siTE}-, or Abd-B^{siTE}-Gal4 were immunostained with anti-Eve, anti-Hox, and anti-mCherry (for dsRFP) or anti-GFP (for dsGFP) antibodies and analyzed as follows. For each of three preparations for each Hox^{siTE}-Gal4 driver, we identified 3 to 5 Eve^{+} cells (U motor

neurons) in 5-6 VNC hemisegments in which the Hox gene was expressed and scored them for co-expression of anti-GFP/mCherry or anti-Hox. If the signal was not above background for either channel, they were scored as non-expressing. The number of Eve^+ cells that were both Hox^+ and GFP/RFP^+ were counted and "Driver Fidelity" was determined by dividing the sum of the cells counted in all three preparations by the total number of Eve^+ cells examined and multiplying by 100. Similarly, we counted cells with mismatched labeling of the following two types: (1) Hox^+ but not GFP/RFP^+ ("false negatives") or (2) GFP/RFP^+ but not Hox^+ ("false positives") and calculated the percentages of each type from the sums across the three animals divided by the total number of Eve^+ cells examined.

Analysis of GNG volume: Four CNS preparations of 5-7 d old adults were immunostained with anti-nc82 antibody (DSHB, Iowa University) for each of the following genotypes: w^{1118};; (control), ;;$Dfd\text{-}Gal4^1$/TM3,Sb, and ;;$Dfd\text{-}Gal4^2$/TM3,Sb (experimental). Each CNS was then imaged by confocal microscopy in 1 μm sections using a Nikon A1-R confocal microscope. Gnathal ganglia (GNG) neuropil was identified according to the criteria of Ito et al. (8) and outlined in each Z-section of each imaged prep using the Freehand tool of Fiji. The outlined areas were then multiplied by the section depth (1 μm) and summed over the entire preparation to obtain the GNG volume for each preparation.

Behavioral Assays

Larval behavior analysis: Larval behavior was analyzed as described previously (9). Briefly, L1 larvae screened for expression of UAS-Cs.Chrimson.mVenus were moved to apple juice caps with either yeast paste (-ATR) or yeast paste with 0.5mM of all-trans

retinol (+ATR) and were raised to third instar. At L3, larvae were transferred to arenas made with 2 mm of 2% agarose in water. Agarose arenas were placed on a circular plexiglass sheet illuminated with 850 nm LEDs (HK-F3528IR60-X from ledlightsworld.com) and behavior was recorded using a Point Grey GS3-U3-41C6NIR camera with a 16 mm Standard Schneider Compact VIS-NIR Lens and a 825 nM high-performance long-pass filter (Edmund Optics, Barrington, NJ). The camera was attached to a Zeiss Stemi 508 dissecting microscope and images were acquired at 10 fps. Optogenetic stimulation was achieved with 1200 lumens of 605 nm light (measured at 50% PWM) using a 1 ½ ft strip of 28 5050SMD LEDs (superbrightleds.com, NFLS-Y30X3-Bk-LC2) mounted on a plexiglass circle (6 in diameter). Larval locomotion was routinely recorded 10 s prior to LED stimulation, 10 s during LED stimulation, and 10 s after stimulation. Stimulating light and camera shutter were controlled with an Arduino Uno microcontroller.

Larval images were processed in Fiji (10). To measure speed of locomotion, the change in position of the larva's posterior tip was calculated as distance (in µm) over time. To measure segment lengths, the multi-point selection tool on ImageJ was used to distinguish segment boundaries and establish regions as shown in Fig. 4A'. The values were exported into MATLAB and segment lengths were calculated for each stimulus condition (for code see doi: 10.6084/m9.figshare.c.7018752).

Adult behavioral analysis: All flies were raised at 18°C to prevent activation of the dTrpA1 channel, which was used for thermogenetic neuronal activation. Experiments were conducted at 18°C or 31°C as indicated in the text. For recording behavioral responses, a 10 µl pipette tip was glued to the dorsal thorax of each fly using Devcon 5

Minute Epoxy and the fly was positioned above a Peltier plate. Plate temperatures were adjusted to yield final temperatures at the fly of 22ºC (inactive condition) or 31ºC (stimulation condition). Behavior was recorded using a Sony NEX-VG10 video camera mounted on an Olympus SZX16 microscope.

Abdominal curvature was measured using the Kappa-Curvature Analysis tool in Fiji (10) from the second or third abdominal segment to the posterior tip. For each animal, abdominal curvature was measured in six video frames—spaced 30 s apart–for the time intervals immediately preceding and immediately following onset of stimulation (i.e. temperature elevation). The mean curvature for each condition was calculated by averaging the six measurements and the ratio of the means (before stimulation vs during stimulation) was used to calculate the normalized curvature observed for each animal. The means and standard deviations of normalized curvatures were calculated across animals of each genotype and the significance of the difference of means from 1 (i.e. the normalized curvature without stimulation) was estimated by t-test.

Supporting Figures

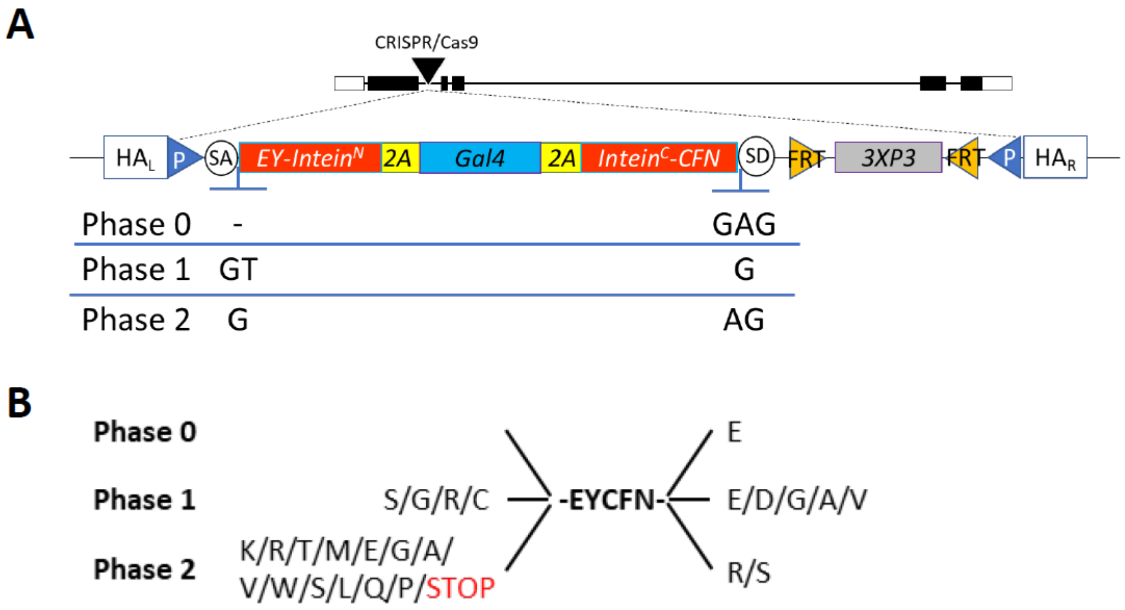

Fig. S1. Design of the siTrojan exon construct using the Cfa split intein.

(A) Schematic of the siTrojan Gal4 constructs, indicating the nucleic acid insertions adjacent to the splice acceptor (SA) and splice donor (SD) sites required to maintain phase for each of the three possible reading frames during protein translation. Choice of reading frame and right and left homology arms (HA_L and HA_R) are determined by the specific intron into which the siTrojan construct is to be inserted by CRISPR/Cas9 genome editing. All other details of the three constructs are the same. Each is flanked by attP (P) sites to permit ΦC31 integrase-mediated cassette exchange. Flippase recognition target (FRT) sites flank a removable 3XP3-RFP selection marker cassette.

(B) Depending on the reading frame of the intron into which the siTrojan construct is inserted and the identity of the codon interrupted, the insertion will lead to the incorporation of a slightly different hexa-or septa-peptide sequence into the native protein at the site of interruption by the intron. All peptides will share the EYCFN sequence of the split intein moieties. All other possible amino acid additions on either side of this penta-peptide are indicated. These depend on the identity of the nucleotide(s) preceding the splice junction in the native gene and the codon formed after splicing in the siTrojan exon. Phase 2 intronic insertions must be avoided if the nucleotides preceding the splice junction are UA, which will occur if a Tyr residue encoded by a UAU or UAC codon has been interrupted. This will lead to insertion of the UAG STOP codon into the protein encoding sequence.

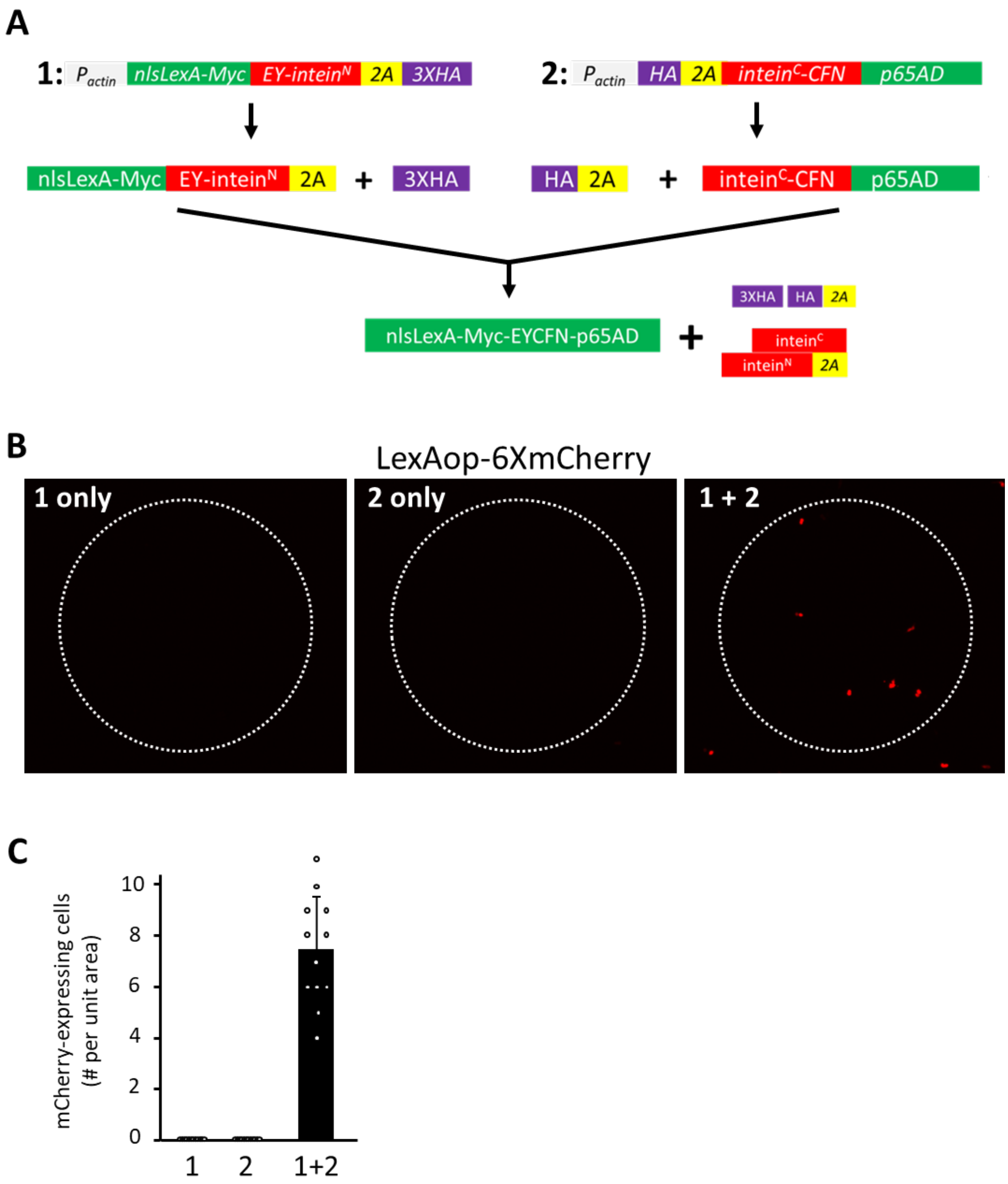

Fig. S2. In vitro demonstration of Cfa split intein efficacy in *Drosophila* cells with a split LexA-p65AD.

(A) Schematic illustrating constructs encoding: (1) the Myc-tagged DNA-binding LexA protein fused to the CfaN split intein moiety followed by T2A and 3 copies of the HA antigen; and (2) the p65 transcription activation domain fused to the CfaC split intein moiety preceded by an HA tag and T2A (top). Translation of these constructs, with attendant ribosomal skipping after translation of the T2A sequences, is predicted to yield the four products shown (middle). The two products at the extreme right and left will be

ligated by action of the Cfa split intein to yield the intact, functional LexA::p65AD transcription factor.

(B) Transfection of *Drosophila* S2 cells in culture with only construct 1 (left) or 2 (middle) does not result in expression of LexAop-6XmCherry, whereas transfection with both constructs 1 and 2 (right) does. This result provides strong evidence that Cfa split intein catalyzes the reconstitution of LexA::p65AD in vitro, and demonstrates that CfaN function is not impaired when the T2A is fused to its C-terminus.

(C) Density of mCherry-positive cells measured in two replicates of the transfection experiment shown in (B). For each condition, cells were counted in six random fields of view equivalent in size to the dotted circles shown in (B) on each of two cell culture plates, and the total was divided by the number of fields counted (n=12, Std dev is indicated).

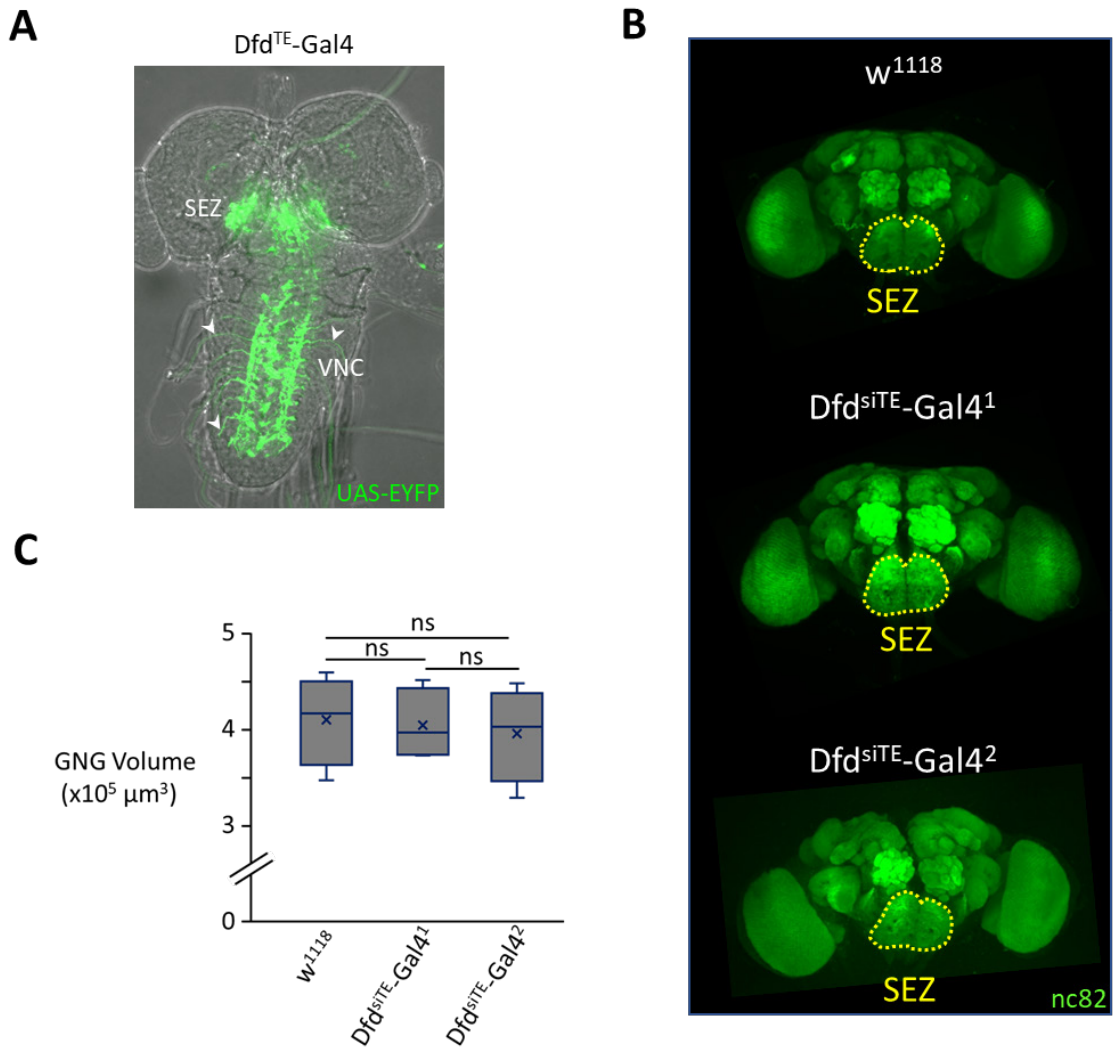

Fig. S3. siTrojan-Gal4 insertions into *Dfd* do not alter SEZ morphology.

(A) The larval CNS expression pattern of the Dfd^{TE}-Gal4 line, made by insertion of a conventional Trojan exon (i.e. TGEM) insertion into site 1 (see Fig. 1F). SEZ expression in this line is similar to that of the Dfd^{siTE}-Gal4 lines made with siTrojan-Gal4 insertions, but consistent labeling of VNC neurons is also observed as can be seen from the lateral axon projections (arrowheads). Similar ectopic labeling was also seen in some Dfd^{siTE}-Gal4^{1} preparations, which have insertions into the same intron. Green, UAS-EYFP

(B) Volume-rendered confocal images of brain neuropil labeling with nc82 antibody for control (w^{1118}) flies and flies heterozygous for siTrojan-Gal4 insertions into the *Dfd* gene at sites 1 and 2 (see Fig. 1F). The SEZ is outlined in yellow and was similar for all genotypes.

(C) Box plots comparing the volume of the gnathal ganglia (GNG, the part of the SEZ in which the *Dfd* gene is expressed) for the each of the three genotypes indicated in (B). GNG volume was calculated from confocal z-stacks using Fiji as described in the

Extended Methods and was not significantly different across genotypes, as determined by t-test (n=4 for each genotype).

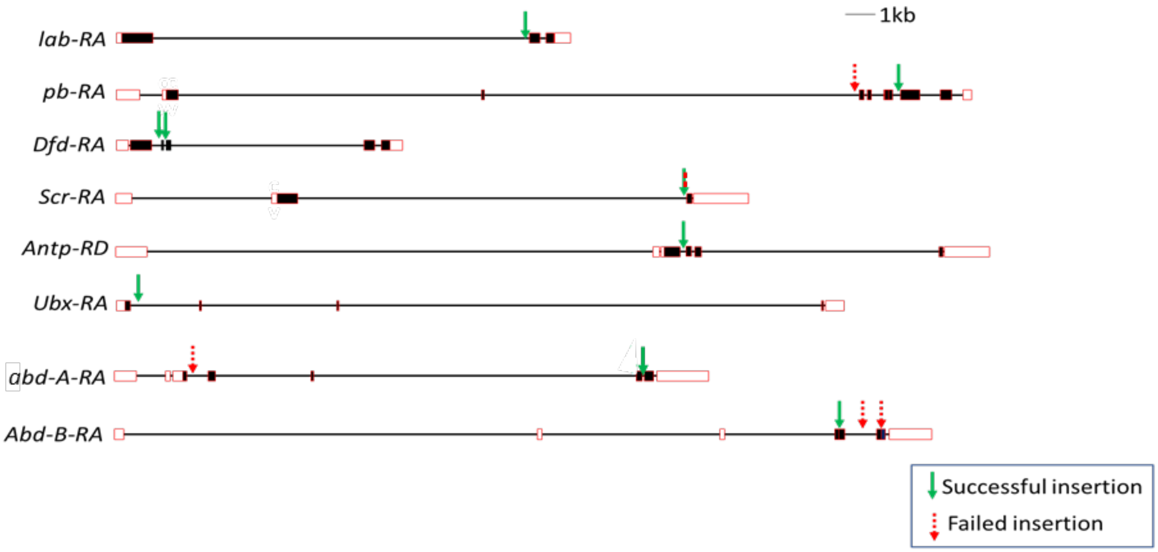

Fig. S4. Intronic sites of insertion of siTrojan exons into *Drosophila* Hox genes

siTrojan-Gal4 insertions were attempted for all eight Hox genes of the fly. Green arrows indicate successful insertions for which stable, viable lines were established. Red arrows indicate attempted insertions that failed to yield viable transformants. Note that for the *Scr* gene, both a green and dotted red arrow are indicated at the same site. No viable siTrojan-Gal4 insertion was obtained at the indicated site, but a viable siTrojan-p65AD insertion was obtained. For reasons not fully understood, Trojan insertions of p65AD tend to be healthier and more viable than insertions of Gal4 and Gal4DBD into the same site. More generally, however, the success or failure of transformation for a given siTrojan insertion is likely to depend on two main things: 1) how efficient the split intein is in the protein context defined by the specific site at which it is inserted, and 2) how well tolerated the insertion peptide incorporated into the full-length protein is at that site after ligation.

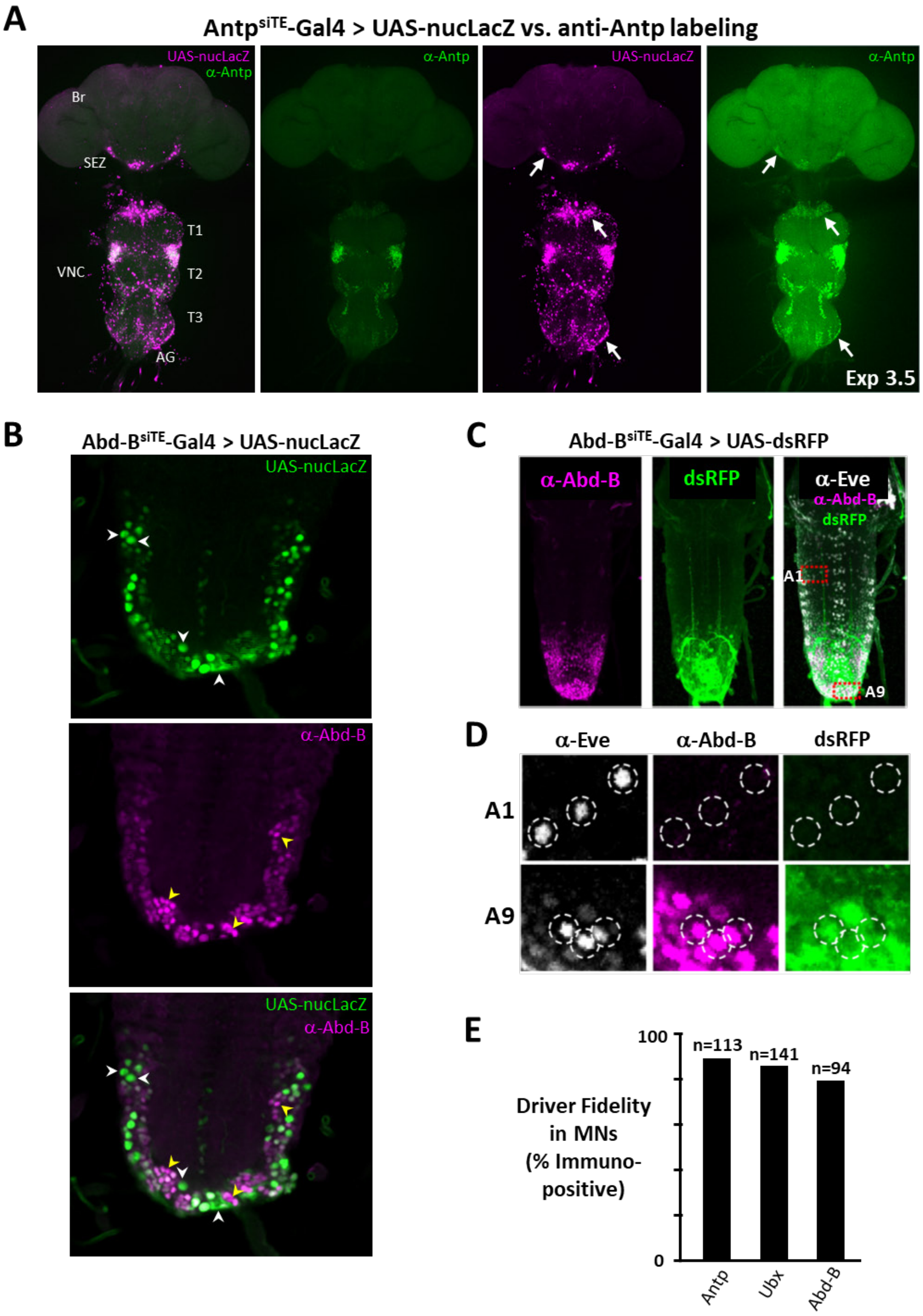

Fig S5. Expression and fidelity of Hox-specific siTrojan drivers.

(A) Expression of Antp^{siTE}-Gal4 in the adult CNS (UAS-nucLacZ, magenta) is strongest at the anterior boundary of T2, consistent with anti-Antp immunoreactivity (α-Antp, green), but it is also enhanced in the labial neuromere of the SEZ, the anterior margin of T1 and the posterior margin of T3 (arrows; 3rd panel, UAS-LacZ). Anti-Antp immunoreactivity in these regions is close to background (2nd panel, α-Antp), but can be seen to be present at higher exposure (4th panel, α-Antp).

(B) Abd-B^{siTE}-Gal4 driven UAS-nucLacZ expression (top, green) and anti-Abd-B immunostaining (middle, magenta) are both restricted to the posterior segments of the L3 CNS, but strength of the two signals is not always well correlated. Although most cells are double-labeled in this Z-section of a confocal stack, strong UAS-LacZ signal doesn't generally coincide with strong anti-Abd-B labeling. In some cases, UAS-LacZ staining is present in the absence of anti-Abd-B labeling (white arrowheads), and in others anti-Abd-B labeling in present in the absence of UAS-LacZ staining (yellow arrowheads).

(C) Anti-Abd-B immunostaining (left, magenta) and Abd-B^{siTE}-Gal4 driven UAS-dsRFP expression (middle, green) are both restricted to the posterior segments of the L1 CNS, but strength of the two signals is not always correlated. The preparation is also immunolabeled for Even-skipped (right, white) and hemisegments in A1 and A9 are outlined in red.

(D) Measuring the degree of overlap between anti-Abd-B immunostaining (middle, magenta) and UAS-dsRFP expression (right, green) in identified Eve^{+} U motor neurons (left, white) from hemisegments A1 and A9 shown in (B). In the posterior segment A9, but not the anterior segment A1, the identified motor neurons are labeled by both anti-Abd-B and anti-mCherry antibodies.

(E) Quantification of overlap (i.e. "Driver Fidelity") of Hox^{siTE}-Gal4 driven reporter expression and corresponding Hox protein immunolabeling in the Eve^{+} U motor neurons of the L1 CNS. Overlap was scored in 5-6 hemisegments in each of three animals as shown in (C) and the percentage of immunoreactive Eve^{+} motor neurons that also expressed UAS-dsRFP or dsGFP is shown for the Antp^{siTE}-, Ubx^{siTE}-, and Abd-B^{siTE}-Gal4 drivers. n, total number of cells analyzed.

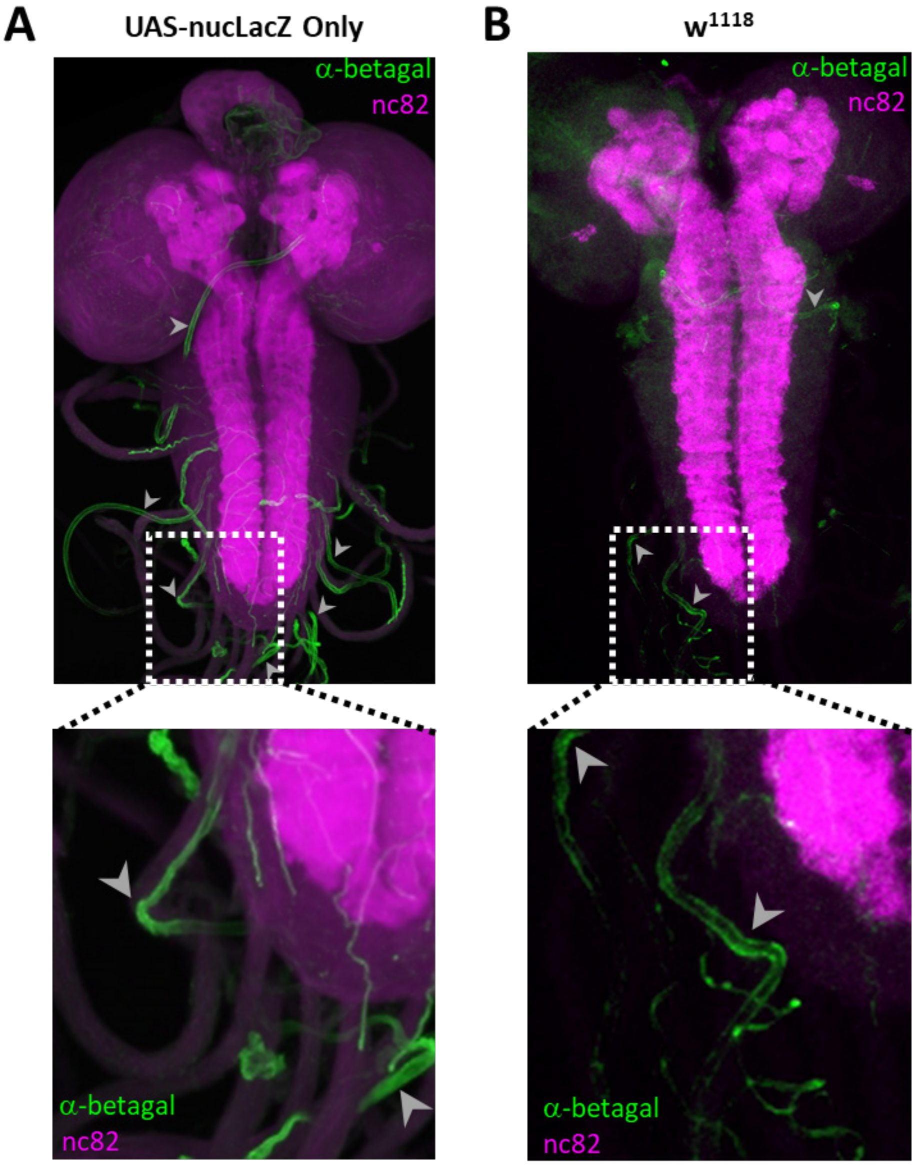

Fig. S6. Gal4- and UAS-nucLacZ-independent labeling of nerves by anti-β-galactosidase antibody

Variable labeling of nerves by anti-β-galactosidase antibody (anti-betagal) was frequently observed in preparations lacking either (A) Gal4 or even (B) UAS-nucLacZ. This labeling was uniform along the length of the nerves rather than isolated to nuclei as is expected for the β-galactosidase reporter encoded by nucLacZ, which has a nuclear localization signal. This uniform nerve labeling, which contrasts with the punctate, nuclear labeling seen in the examples indicated by yellow arrows in Fig. 2B, thus appears to be an artifact of the anti-betagal staining although the source of its variability in terms of the number of nerves labeled—both across and within genotypes—remains unclear. (A) Anti-betagal (green) labeled larval CNS (top) from an animal with the genotype: w;+/(Sp or Cyo);UAS-nucLacZ/TM3,Sb. Inset (bottom) shows an enlarged view of the

dotted region. Gray arrowheads, examples of labeled nerves. Neuropil is stained with anti-Brp antibody nc82 (magenta)

(B) Anti-betagal labeled larval CNS (top) from animal with the genotype w^{1118}. Inset (bottom) and labels are as described in (A).

Supporting Table S1: Previously Generated Hox Gene Drivers and Hemidrivers

Hox Gene	Driver Type	Method of Generation	Publication
**lab**	Gal4	promoter fusion, p-element	(11)
**pb**	Gal4	promoter fusion, p-element	(12)
	Gal4-T2A	CRISPR-mediated ATG knock in	(13)
**Dfd**	Gal4-T2A	CRISPR-mediated ATG knock in	(13)
	Gal4DBD	Start codon knock-in, ends-out HR	(14)
	p65AD	Start codon knock-in, ends-out HR	(14)
	LexA	Start codon knock-in, ends-out HR	(14)
**Scr**	LexA	Start codon knock-in, ends-out HR	(14)
	Gal4-T2A	CRISPR-mediated ATG knock in	(13)
**Antp**	Gal4	promoter-fusion, p-element	(15)
	Gal4	p-element swap of LacZ line	(16)
	Gal4	Start codon knock-in, ends-out HR	(14)
**Ubx**	Gal4	p-element swap of LacZ line	(17)
	Gal4	p-element swap of LacZ line	(18)
	Gal4DBD	enhancer fusion, attP2	(19)
**abd-A**	Gal4	p-element swap of LacZ line	(20)
**Abd-B**	Gal4	p-element swap of LacZ line	(18)
	Gal4DBD	p-element swap of LacZ line	(14)

Supporting Table S2: Comparison of Methods of Gene-specific Gal4 and LexA Driver Line Generation and their Application to Hox Genes

Driver Type	Fidelity	RMCE Ready	Mutagenic	Isoform Specificity	Intron Dependent	Hox Gene Lines
**Enhancer/ Promoter fusion**	Variable	No	No	High	No	lab-Gal4 (11); pb-Gal4 (12); Antp-P1-Gal4 (15)
**Enhancer-trap**	Variable	No	Variable	Variable	No	Antp-Gal4 (16); Ubx-Gal4 (17, 18); abd-A-Gal4 (20); Abd-B-Gal4 (18)
**Knock-in (with gene replacement)**	High	Yes	Very (null)	High	No	Dfd-LexA, Scr-LexA, Antp-Gal4 (14)
**Knock-in (with co-expression)**	High	No	No	High	No	pb-Gal4, Dfd-Gal4, Scr-Gal4 (13)
**Trojan exon (TGEM/ CRIMIC)**	High	Yes	Very (truncation)	Low	Yes	Dfd^{TE}-Gal4 (this paper)
**siTrojan exon**	High	Yes	Variable (6-7 peptide insertion; possible persistent truncation products)	Low	Yes	lab^{siTE}-Gal4, pb^{siTE}-Gal4, Dfd^{siTE}-Gal4, $Antp^{siTE}$-Gal4, Ubx^{siTE}-Gal4, $abd\text{-}A^{siTE}$-Gal4, $Abd\text{-}B^{siTE}$-Gal4 (this paper)

Gene-specific driver lines have been generated using various methods for capturing the regulatory elements responsible for gene expression. The degree to which regulatory information is captured ("Fidelity") varies with method and each method has its own advantages and disadvantages as indicated in the table columns. Some support recombinase-mediated cassette exchange ("RMCE ready") for easy transgene replacement, some necessarily (or can) mutate the targeted gene ("Mutagenic"), while others necessarily target specific isoforms of the gene ("Isoform Specificity") or require the gene to contain an intron separating two coding exons ("Intron Dependent"). Hox gene lines generated using the various methods are indicated. Since no one method will generally meet all requirements for a gene-specific driver, having multiple available techniques is in itself an advantage.

Supporting Movies

Movie S1 (separate file). Head lifting in response to activation of Scr^{siTE}∩VGlut neurons. Speed: 3X.

Movie S2 (separate file). Foreleg grooming by a decapitated fly in response to activation of Scr^{siTE}∩ChaT neurons. Speed: 3X.

SI References

1. F. Diao *et al.*, Plug-and-Play Genetic Access to Drosophila Cell Types using Exchangeable Exon Cassettes. *Cell Reports* **10**, 1410-1421 (2015).
2. A. J. Stevens *et al.*, Design of a Split Intein with Exceptional Protein Splicing Activity. *J Am Chem Soc* **138**, 2162-2165 (2016).
3. F. Q. Diao, B. H. White, A Novel Approach for Directing Transgene Expression in Drosophila: T2A-Gal4 In-Frame Fusion. *Genetics* **190**, 1139-U1356 (2012).
4. X. J. Ren *et al.*, Optimized gene editing technology for Drosophila melanogaster using germ line-specific Cas9. *Proceedings of the National Academy of Sciences of the United States of America* **110**, 19012-19017 (2013).
5. K. J. Venken *et al.*, MiMIC: a highly versatile transposon insertion resource for engineering Drosophila melanogaster genes. *Nat Methods* **8**, 737-743 (2011).
6. H. J. Luan, F. Q. Diao, N. C. Peabody, B. H. White, Command and Compensation in a Neuromodulatory Decision Network. *Journal of Neuroscience* **32**, 880-889 (2012).
7. E. S. Heckscher *et al.*, Atlas-builder software and the eNeuro atlas: resources for developmental biology and neuroscience. *Development* **141**, 2524-2532 (2014).
8. K. Ito *et al.*, A Systematic Nomenclature for the Insect Brain. *Neuron* **81**, 755-765 (2014).
9. C. C. Wreden *et al.*, Temporal Cohorts of Lineage-Related Neurons Perform Analogous Functions in Distinct Sensorimotor Circuits. *Current Biology* **27**, 1521-+ (2017).
10. J. Schindelin *et al.*, Fiji: an open-source platform for biological-image analysis. *Nature Methods* **9**, 676-682 (2012).
11. F. Hirth *et al.*, Functional equivalence of Hox gene products in the specification of the tritocerebrum during embryonic brain development of Drosophila. *Development* **128**, 4781-4788 (2001).
12. C. Benassayag *et al.*, Evidence for a direct functional antagonism of the selector genes proboscipedia and eyeless in Drosophila head development. *Development* **130**, 575-586 (2003).
13. A. Auradkar, E. A. Bulger, S. Devkota, W. McGinnis, E. Bier, Dissecting the evolutionary role of the Hox gene proboscipedia in Drosophila mouthpart diversification by full locus replacement. *Sci Adv* **7** (2021).
14. J. H. Simpson, Rationally subdividing the fly nervous system with versatile expression reagents. *Journal of Neurogenetics* **30**, 185-194 (2016).
15. D. J. Andrew, M. A. Horner, M. G. Petitt, S. M. Smolik, M. P. Scott, Setting Limits on Homeotic Gene-Function - Restraint of Sex-Combs-Reduced Activity by Teashirt and Other Homeotic Genes. *Embo Journal* **13**, 1132-1144 (1994).
16. B. S. Emerald, S. M. Cohen, Spatial and temporal regulation of the homeotic selector gene Antennapedia is required for the establishment of leg identity in Drosophila. *Developmental Biology* **267**, 462-472 (2004).
17. S. K. Pallavi, L. S. Shashidhara, Egfr/Ras pathway mediates interactions between peripodial and disc proper cells in Drosophila wing discs. *Development* **130**, 4931-4941 (2003).
18. L. de Navas, D. Foronda, M. Suzanne, E. Sanchez-Herrero, A simple and efficient method to identify replacements of P-lacZ by P-Gal4 lines allows obtaining Gal4

insertions in the bithorax complex of Drosophila. *Mechanisms of Development* **123**, 860-867 (2006).
19. A. R. Issa, J. A. C. Menzies, A. Padmanabhan, C. R. Alonso, A novel post-developmental role of the Hox genes underlies normal adult behavior. *Proceedings of the National Academy of Sciences of the United States of America* **119** (2022).
20. B. Hudry, S. Viala, Y. Graba, S. Merabet, Visualization of protein interactions in living Drosophila embryos by the bimolecular fluorescence complementation assay. *Bmc Biology* **9** (2011).
